# Identification of genetics and hormonal factors involved in *Quercus robur* root growth regulation in different cultivation system

**DOI:** 10.1186/s12870-024-04797-z

**Published:** 2024-02-20

**Authors:** Paulina Kościelniak, Paulina Glazińska, Jacek Kęsy, Joanna Mucha, Marcin Zadworny

**Affiliations:** 1 Department of Ecology, Institute of Dendrology, Polish Academy of Sciences, 62-035 Kórnik, Poland; 2 Department of Plant Physiology and Biotechnology, Faculty of Biological and Veterinary Sciences, Nicolaus Copernicus University, 87-100 Toruń, Poland; 3 Faculty of Forestry and Wood Technology, Poznan University of Life Sciences, Wojska Polskiego 71a, 60-625 Poznań, Poland

**Keywords:** Gene, Hormone, Oak, RNA-Seq, Root, Transcriptome

## Abstract

Understanding the molecular processes and hormonal signals that govern root growth is of paramount importance for effective forest management. While Arabidopsis studies have shed light on the role of the primary root in root system development, the structure of root systems in trees is considerably more intricate, posing challenges to comprehend taproot growth in acorn-sown and nursery-cultivated seedlings. In this study, we investigated *Quercus robur* seedlings using rhizotrons, containers, and transplanted containers to rhizotrons, aiming to unravel the impact of forest nursery practices on processes governing taproot growth and root system development. Root samples were subjected to RNA-seq analysis to identify gene expression patterns and perform differential gene expression and phytohormone analysis. Among studied cultivation systems, differentially expressed genes (DEGs) exhibited significant diversity, where the number of co-occurring DEGs among cultivation systems was significantly smaller than the number of unique DEGs in different cultivation systems. Moreover, the results imply that container cultivation triggers the activation of several genes associated with linolenic acid and peptide synthesis in root growth. Upon transplantation from containers to rhizotrons, rapid enhancement in gene expression occurs, followed by gradual reduction as root growth progresses, ultimately reaching a similar expression pattern as observed in the taproot of rhizotron-cultivated seedlings. Phytohormone analysis revealed that taproot growth patterns under different cultivation systems are regulated by the interplay between auxin and cytokinin concentrations. Moreover, the diversification of hormone levels within the root zone and cultivation systems allows for taproot growth inhibition and prompt recovery in transplanted seedlings. Our study highlights the crucial role of hormone interactions during the early stages of taproot elongation, influencing root system formation across.

**Supplementary Information:**

The online version contains supplementary material available at 10.1186/s12870-024-04797-z.

## Introduction

Periodic lowering of groundwater levels caused by prolonged droughts, and subsequent soil drying, have been linked to the premature decline of oak stands [1–4]. In naturally-regenerated oak stands, trees can cope with drought conditions by developing a robust, deep root system that accesses water from deeper soil layers. This facilitates water uptake from descending water tables and enhances the trees' ability to survive prolonged water shortages [5, 6]. As a result, seedlings with deep-rooted systems have a higher chance of surviving severe droughts compared to those with shallow root systems. Deeper rooting also improves the survival of bareroot seedlings [7]. Unfortunately, the most commonly used regeneration forestry practices, such as undercutting the taproot, which does not regenerate [8], alter root architecture and result in abundant but shallow oak root systems. These altered root systems limit above-ground development and increase vulnerability to drought, similar to the effects of coppicing [9, 10]. Root pruning management practices disrupt the natural process of further root system development and may contribute to reduced oak survival during long-term drought episodes. However, nursery practices for container-grown seedlings could enhance seedling survival after outplanting, even under drought stress, by improving root morphology and depth. Unfortunately, despite having drainage openings at the bottom of containers, root air pruning occurs, limiting outward root growth and injuring the taproots [10]. Altering seedling development in containers through taproot injury could make the seedlings more susceptible to unfavorable environmental conditions [11]. However, a decline in root system quality does not always occur, as taproots were observed within 55% of containerized seedlings after their outplanting into the experimental pots [10]. Nevertheless, it is worth noting that not all taproots grow out of the container; some mechanism restricts taproot elongation before reaching the container's bottom. The ability to achieve a natural-like root system in a fraction of containerized seedlings could significantly improve their resistance to prolonged water shortages and enhance the overall quality of planting materials from containerized nurseries. However, information on the relationship between nursery treatments and subsequent growth, as well as the overall vitality of oak stands, is limited for seedlings produced in containers [12]. Promoting taproot regrowth in containerized seedlings upon outplanting in forests could enhance their access to water resources and positively influence oak development, increasing their ability to withstand long-term drought episodes. Thus, there is a pressing need to identify the factors that control taproot growth, cessation of growth, and regrowth.

Root growth and development are influenced by internal genetic and physiological factors as well as environmental conditions. However, most research on seedling quality in container nurseries has primarily focused on phenotypical or physiological aspects, neglecting the molecular determinants that govern root growth and development [13–15]. The level and activity of molecular and physiological factors can lead to developmental and structural diversity in seedlings growing under different cultivation systems in two ways: 1) internal signaling that shapes root growth patterns, and 2) coordination of root growth in response to environmental stimuli. Consequently, the regulation of taproot growth is multidimensional, influenced by signaling and gene expression levels that directly impact root growth dynamics. Studying these processes, from sensing environmental cues, such as those in containerized transplants, to molecular and physiological signaling cascades that initiate taproot growth (which has been inhibited in containers), presents a significant challenge. Nevertheless, fundamental questions about the role of hormonal signals and molecular processes in regulating root architecture, particularly taproot growth, remain unanswered.

Hormones are well-documented regulators of root development and growth. For instance, auxins are involved in initiating root formation [16], but high concentrations of auxins can inhibit root elongation [17]. Cytokinins control the cessation of root growth, preventing excessive lateral root growth [18], while ethylene inhibits root elongation, and its production is stimulated by auxins [19]. However, ethylene also inhibits auxin transport [20]. The interaction and cross-talk among these hormones regulate root growth and determine taproot dominance. For instance, ethylene's inhibition of auxin transport leads to auxin accumulation in primary root tips, effectively inhibiting root elongation [21]. Cytokinins, by modulating ethylene levels through the regulation of ACS (aminocyclopropane-1-carboxylic acid synthase) gene family members, are also involved in root elongation inhibition [22]. Detailed knowledge of how these hormonal signals cooperate can shed light on components of plant hormonal responses that inhibit and allow regrowth of roots. This is further supported by the observation of a wide variety of hormonal responses between different natural *Arabidopsis thaliana* (L.) accessions [23] and species, such as *Brachypodium distachyon*, which displays profound differences compared to Arabidopsis [24]. Understanding the processes that control taproot function in containerized oak seedlings requires the identification and characterization of endogenous factors regulating taproot growth and inhibition. This information is crucial for developing efficient strategies to promote taproot regrowth in containerized oak seedlings after transplantation into the field.

The objective of this study was threefold: 1) to analyze temporal changes in gene expression patterns during taproot elongation in an experimental system that mimics natural conditions, nursery containers, and transplanted containerized seedlings; 2) to investigate hormone control of root development during elongation in different nursery cultivation systems; and 3) to determine the specificity of the expression profile among different tissues, specifically the meristematic and elongation zones of the taproot and the meristematic zone of the lateral root. To achieve these goals, we conducted RNA-seq to comprehensively profile the taproot (meristematic and elongation zones) and lateral root transcriptomes. Additionally, we analyzed plant hormones to compare their activity in different nursery cultivation systems. Understanding how plants integrate internal and external signals to regulate root growth is crucial for tree cultivation. Therefore, we also discuss the implications of specific signals and signaling pathways that regulate the molecular process of taproot growth at the forest management level.

## Materials and methods

### Plant material, cultivation and sample collection

**Fig. 1:**
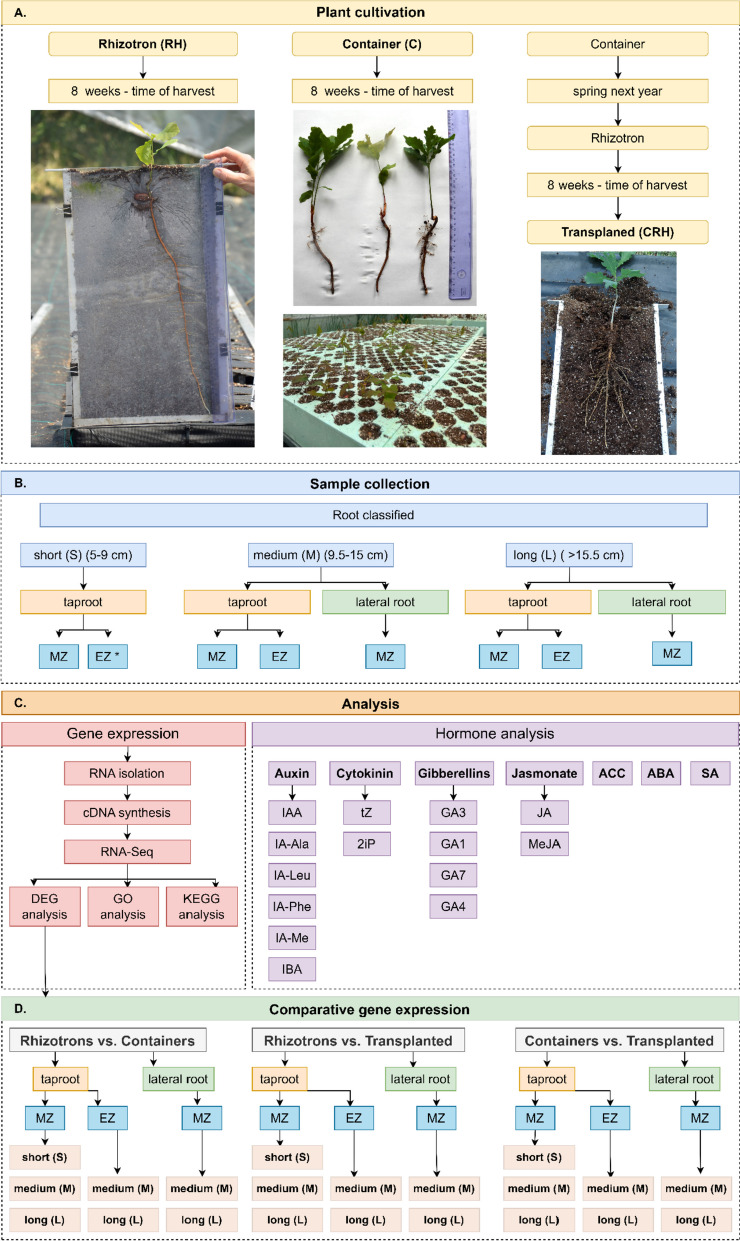
General workflow of experimental steps **A**) plant cultivation **B**) sample collection, **C**) analysis used in research (gene expression and hormone analysis) and **D**) samples used in comparative gene expression. Note that the differences in taproot tip morphology between rhizotron and container seedlings were clearly visible when the seedlings were approximately 10 weeks old (Fig. 1A), although differences in taproots length occurred at least 2 weeks before- at the time of harvest. At that time (taproots of rhizotron seedlings were longer (23 cm on average) than those of container seedlings on average 13.7 cm). Note also the similarity in taproot tip morphology between rhizotron and transplanted seedlings (Fig. 1A). MZ – meristematic zone; EZ – elongation zone; DEG—Differentially expressed genes; GO – Gene Ontology; KEGG – Kyoto Encyclopedia of Genes and Genomes; IAA – indole-3-acetic acid; IBA – indole-3-butyric acid; IA-Ala – indole-3-acetyl-L-alanine; IA-Leu – indole-3-acetyl-L-leucine; IA-Phe – indole-3-acetyl-L-phenylalanine; IA-ME – indole-3-acetyl-L-methionine; tZ – trans-Zeatin; 2iP –N6-(2-Isopentenyl)adenine; GA3, GA1, GA7, GA4 – gibberellins 3,1,7,4; JA – jasmonic acid; MeJA – methyl jasmonate; ACC—1-aminocyclopropane-1-carboxylic acid; ABA – abscisic acid; SA – salicylic acid. An asterisk (*) denotes a research variant for which the transcriptome was not sequenced

In this study, we utilized roots from *Quercus robur* L. seedlings grown under various cultivation systems, and acorns were purchased from a storehouse in Jarocin of the National State Forests (Jarocin forest district, Poland). The experiment took place in a large, semi-closed, and foil greenhouse situated in the Institute of Dendrology, Polish Academy of Sciences, Kórnik, Poland. Three experimental nursery cultivation systems were employed: container, rhizotron, and transplanted (wherein the seedlings were initially grown in containers and later transferred to rhizotrons). The seedlings were cultivated within transparent rhizotron chambers (30 × 50 cm) and containers (180 mm high, 50 mm wide, 0.275 dm^3^). The growing medium used for both systems consisted of a peat and perlite mixture (volumetric proportion of 5:1), supplemented with dolomite for deacidification and slow-release fertilizer (Osmocote 15–9-12–2 N-P-K-Mg, with trace nutrients) at a rate of 2.5 kg/m3. The rhizotron structure comprised two plexiglass plates separated by 2–3 cm and secured with sturdy plastic tubing to ensure adequate root space. Drainage measures were incorporated at the bottom of each rhizotron to prevent waterlogging. The use of rhizotrons enabled non-invasive monitoring of root growth in the same seedlings over time, without disrupting the root system. After 8 weeks of growth, container (C) and rhizotron (RH) seedlings were harvested. The transplanted (CRH) seedlings, however, were grown in containers for the first year, then transplanted into rhizotrons for 8 weeks, and harvested in a comparable period after planting as the seedlings from both the containers and the rhizotrons (Fig. 1A). Taproots were measured and classified based on their length into three categories: S—short (5–9 cm), M—medium (9.5–15 cm), and L—long (> 15.5 cm). In this study, we focused on the tips of taproots, dividing them into the meristematic zone (TR) and elongation zone (EZ) of the taproot (Fig. 1B). Based on the previously done anatomical characteristics (data now showed), specific root segments were categorized into meristematic and elongation zone. Lateral roots (LR) were obtained from corresponding lengths of the taproot, namely medium (MLR) and long (LLR), and consisted only of root tips of lateral roots with the meristematic zone. Lateral roots were absent when the taproot length was short [25]. Moreover, the average taproot length varied between cultivation systems, resulting in an average length of 23 cm (± 1.4SE) for rhizotron seedlings and 13.7 (± 0.2SE) for containers seedlings within the 'long root categories' at harvest. In September, most of the taproots of the containers seedlings were in the 13–14 cm length range, whereas many of the taproots of the rhizotron seedlings were 50 cm long and reached the bottom of the rhizotron.

For RNA-seq analysis, we extracted total RNA from the roots of seedlings grown in different cultivation systems using Ribospin (GeneAll Biotechnology, Seoul, South Korea). Subsequently, we constructed a cDNA library with a TruSeq Stranded mRNA LT Sample Prep Kit and sequenced it on a NovaSeq platform (Illumina, San Diego, CA, USA) in the 150 bp PE mode. The expression of selected genes after NGS sequencing was validated through RT-qPCR reactions. The data discussed in this publication have been deposited in NCBI GEO and can be accessed through GEO Series accession number GSE181860 and the OakRootRNADB database [25]. Further information about seedling cultivation, sample collection, sequencing, and validation of sequencing results via RT-qPCR is available in detail in the work by Kościelniak et al. [25].

### Identification, functional and pathway enrichment for DEGs

We analyzed differentially expressed genes (DEGs) to identify variations in gene expression patterns in roots when growing in different cultivation systems. The gene abundances for each sample were estimated using the expectation–maximization method, specifically the RSEM algorithm, and expressed as FPKM (Fragments Per Kilobase Of Exon Per Million Fragments Mapped). The differential gene expression analysis was performed at the transcript level using DESeq2, as described by Love et al. [26]. Genes with a fold change (log2FC) greater than 1.5 and a statistically significant *p*-value < 0.05 were considered differentially expressed. Further analysis of the identified DEGs was carried out using the Gene Ontology (GO) and Kyoto Encyclopedia of Genes and Genomes (KEGG) databases. GO enrichment analyses were performed using the Trinotate-Trinotate-v3.2.2 package script. The GO analysis was performed at the non-ancestral level, which is similar to the ancestral level but excludes ancestral terms from the analysis. A search was conducted to identify significantly enriched GO terms, which were subsequently mapped. To update obsolete terms, data from the AmiGO database (amigo.geneontology.org) were utilized. Some terms in the output were marked as "none" because they had an "obsolete" status in the database. The background for the analysis comprised all the identified genes obtained using Trinotate v 3.0.2. The result files contained terms that were significantly enriched or less frequent than statistically depleted terms, with a *p*-value < 0.05. Similarly, the identified KEGG pathways underwent the same analysis, and the results were filtered to include only those terms derived from plants.

### Plant hormone analysis

Phytohormone levels were measured in the same root zones as used for transcriptome analysis, but in a different batch of tissues using the QuEChERS method (quick, easy, cheap, effective, rugged, and safe) with deuterated internal standards (Fig. 1C). This method utilizes phase separation with acetonitrile and phytohormone extraction on C18 SPE columns (BAKERBOND Octadecyl spe™, Avantor, USA) designed to selectively bind and then dissociate the desired compounds [27]. The preparation of plant material followed the protocol used in the experiment of Pu et al. [28] with necessary modifications. Quantification was done using high-performance liquid chromatography coupled with mass spectrometry (UHPLC-MS/MS) with a Shimadzu Nexera XR UHPLC system (Shimadzu, Kyoto, Japan) and a triple quadrupole mass spectrometer detector (LCMS-8045, Shimadzu). Analyses were carried out using a BAKERBOND Octadecyl spe™ C18 column (Avantor, USA). Chromatographic separation was achieved on an Ascentis Express C18 column (2.7 μm, 100 × 2.1 mm, Supelco, Bellefonte, PA, USA), maintained at 35 °C, using 0.1% formic acid in water (mobile phase A) and methanol with 0.1% formic acid (mobile phase B) at a flow rate of 0.35 mL min^−1^. The gradient started at 35% B, then increased linearly to 90% B over the next 4 min, and finally to 100% B over the next 2 min. Individual phytohormones were identified based on the decay of selected ions using a sensitive and selective mode of quantitative analysis, observing selected fragmentation reactions (MRM—multiple reaction monitoring). The recorded MRM values for individual hormones in positive ( +) or negative (-) ionization are shown in Table S1. Data were analyzed using LabSolutions software 5.8 (Shimadzu, Kyoto, Japan). Log_10_ transformation of data was performed to ensure normal data distribution and homogeneous variances across treatments. Outliers were replaced by the average of valid measurements. The main effect of cultivation systems was determined using one-way ANOVA, and differences between mean values were analyzed using a post-hoc Tukey’s HSD test. Statistical significance was considered at P ≤ 0.05. All analyses were conducted using JMP Pro 13.

## Results

### Identification, functional and pathway enrichment for the DEG

In this study, we conducted an analysis of differentially expressed genes (DEGs) in the meristematic zone and elongation zone of taproots, as well as in the meristematic zone of lateral roots originating from roots cultivated in various types of cultivation systems. The logic and pattern of sample comparison used for comparative gene expression analysis is shown in Fig. 1D. This analysis allowed us to identify genes involved in the development of both root types within their respective growth zones between different cultivation systems. Additionally, we examined the differences between gene expression at various stages of root elongation shortly after emergence. Subsequently, we performed an enrichment analysis of the GO and KEGG pathways for roots from different cultivation systems. The purpose of this analysis was to investigate how the growth and development of roots in different cultivation systems influence the gene expression of taproots and lateral roots.

#### Comparative gene expression analyses of roots from rhizotrons and containers

**Fig. 2:**
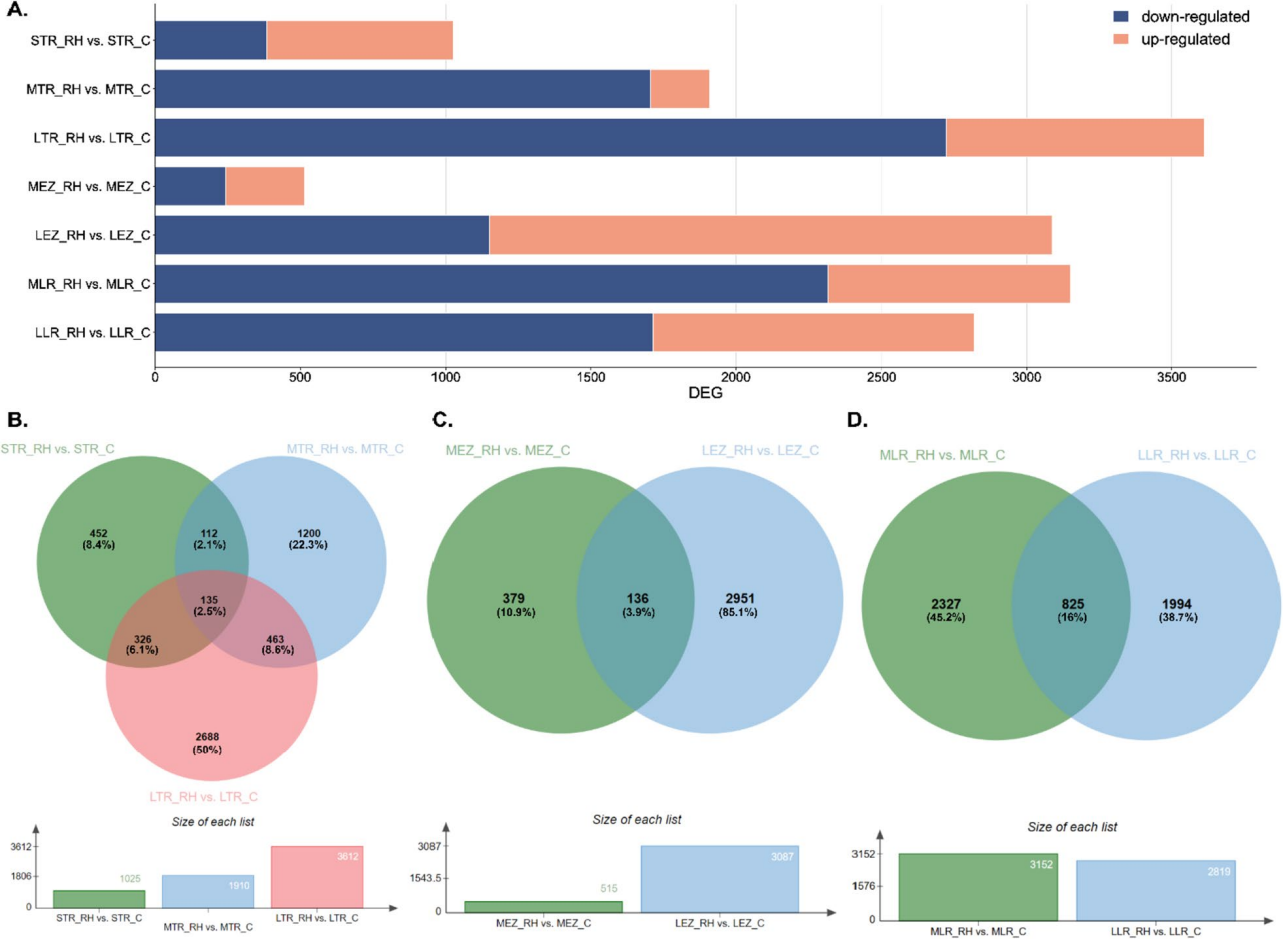
**A**) Comparison of the number of differentially expressed genes (DEGs) in roots between rhizotron and container systems. Venn diagrams showing DEGs identified between **B**) short, medium, and long tips of taproots; **C**) elongation zone from medium and long taproots; **D**) and lateral roots harvested from medium and long taproots based on Bardou et al. (2014) [29]. Annotations with log2FC > 1.5 were used for the analysis of DEGs

In our study, conducted on oak roots grown in rhizotrons and containers, we identified a total of 16,120 differentially expressed genes (DEGs). Among these, 10,227 DEGs were significantly down-regulated, while 5,883 genes were significantly up-regulated in roots growing in rhizotrons. The number of DEGs unique for each comparison is shown in the Venn diagram shown in Fig. 2B-D. This indicates that the up-regulation of gene expression increased much more frequently than down-regulation within roots growing in the container system (Fig. 2A). Within the meristematic zone of the taproot, we observed 135 DEGs (Fig. 2A) that were differentially expressed across all root length categories. This suggests that their expression changes were not solely dependent on root length, possibly implying that different cultivation conditions were the primary influencing factor for gene expression variation. A comparable number of DEGs occurred in the elongation zone (136, Fig. 2C). The effect of elongation on the number of DEGs was relatively consistent in both the meristematic and elongation zones. The number of DEGs was highest when the taproots were longer during growth in containers compared to rhizotrons. This stage was also marked by a significant occurrence of DEGs specific only for the long taproot (Fig. 2B-C). Thus, as the root grew, the number of DEGs increased in the container system. Furthermore, differences in the number of DEGs were also observed when comparing lateral roots. Lateral roots from containerized seedlings displayed a slight decrease in comparison to lateral roots harvested from rhizotrons (Fig. 2A).

**Fig. 3:**
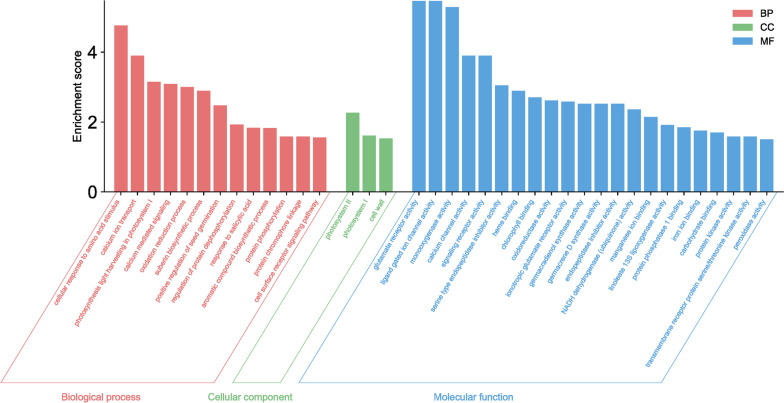
The GO enrichment analysis for the meristematic and elongation zones of taproots with different lengths, and the meristematic zone in lateral roots within the rhizotron and container comparison. Enrichment scores are represented as -log10(FDR). Enriched GO terms were selected based on an enrichment score > 1.5

The functional analysis of these genes revealed their involvement in various metabolic pathways, including amino acid processing, ion transport, regulation of developmental processes, biosynthesis of secondary metabolites, and cell wall construction (Fig. 3).

**Fig. 4:**
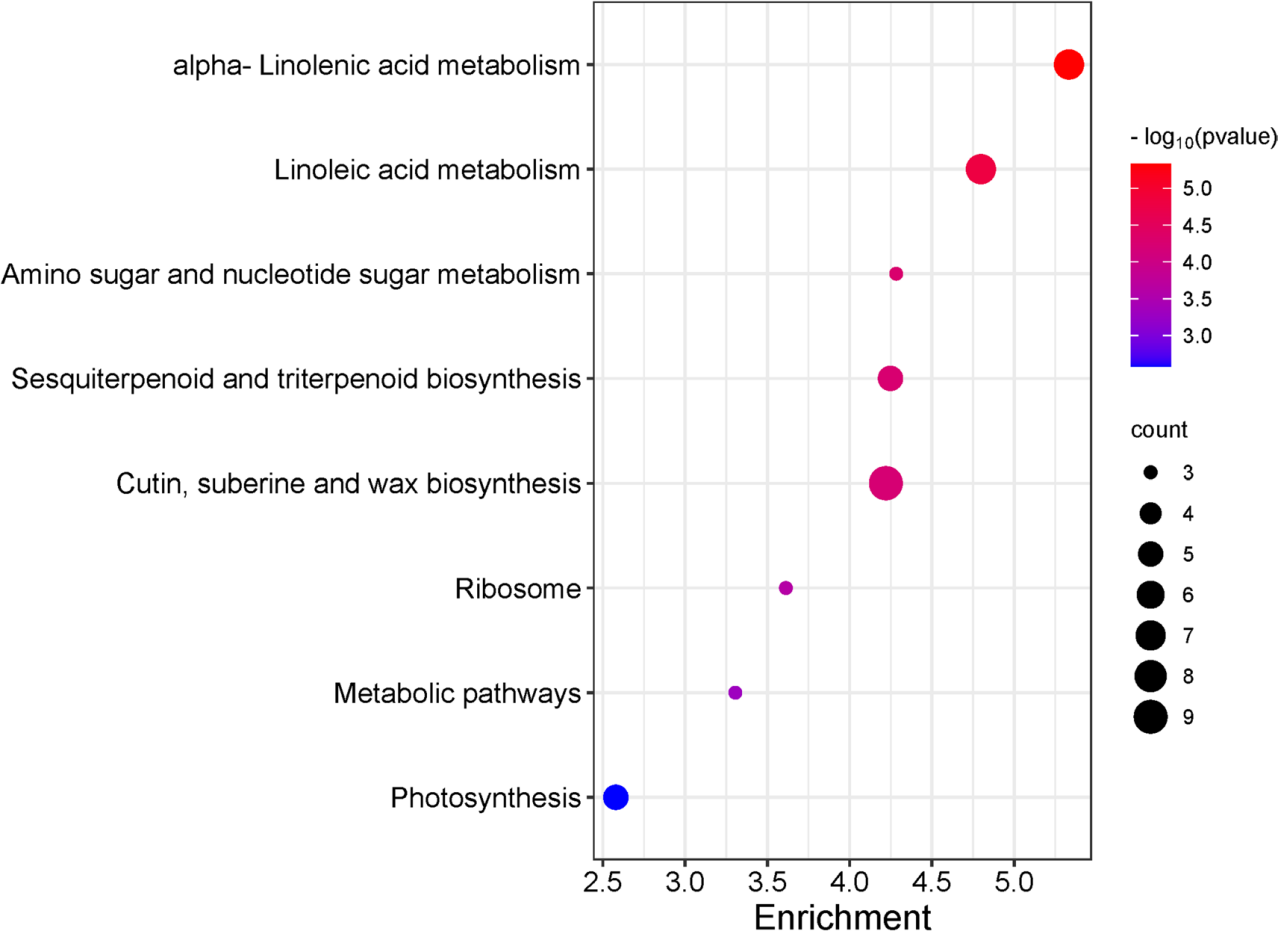
KEGG enrichment analysis of differentially expressed genes (DEGs) in taproot and lateral roots among rhizotron and container cultivation systems. The degree of significance of the enrichment of DEGs in a pathway is indicated by -log10 (*p*-value). Circle size represents the number of genes. The color gradient from blue to red indicates low to high enrichment significance [30]

Next, we conducted KEGG pathway analysis of the differentially expressed genes (DEGs) to gain a deeper understanding of both metabolic and signal transduction pathways. Figure 4 illustrates the top eight enriched pathways. Among the identified KEGG enrichment pathways, we selected only those with a false discovery rate (FDR) < 1.5. The largest number of metabolites was assigned to groups such as linoleic acid metabolism, amino sugar and nucleotide sugar metabolism, sesquiterpenoid and triterpenoid biosynthesis, and cutin, suberine, and wax biosynthesis.

#### Gene expression changes within roots between in rhizotron and transplanted comparison

**Fig. 5:**
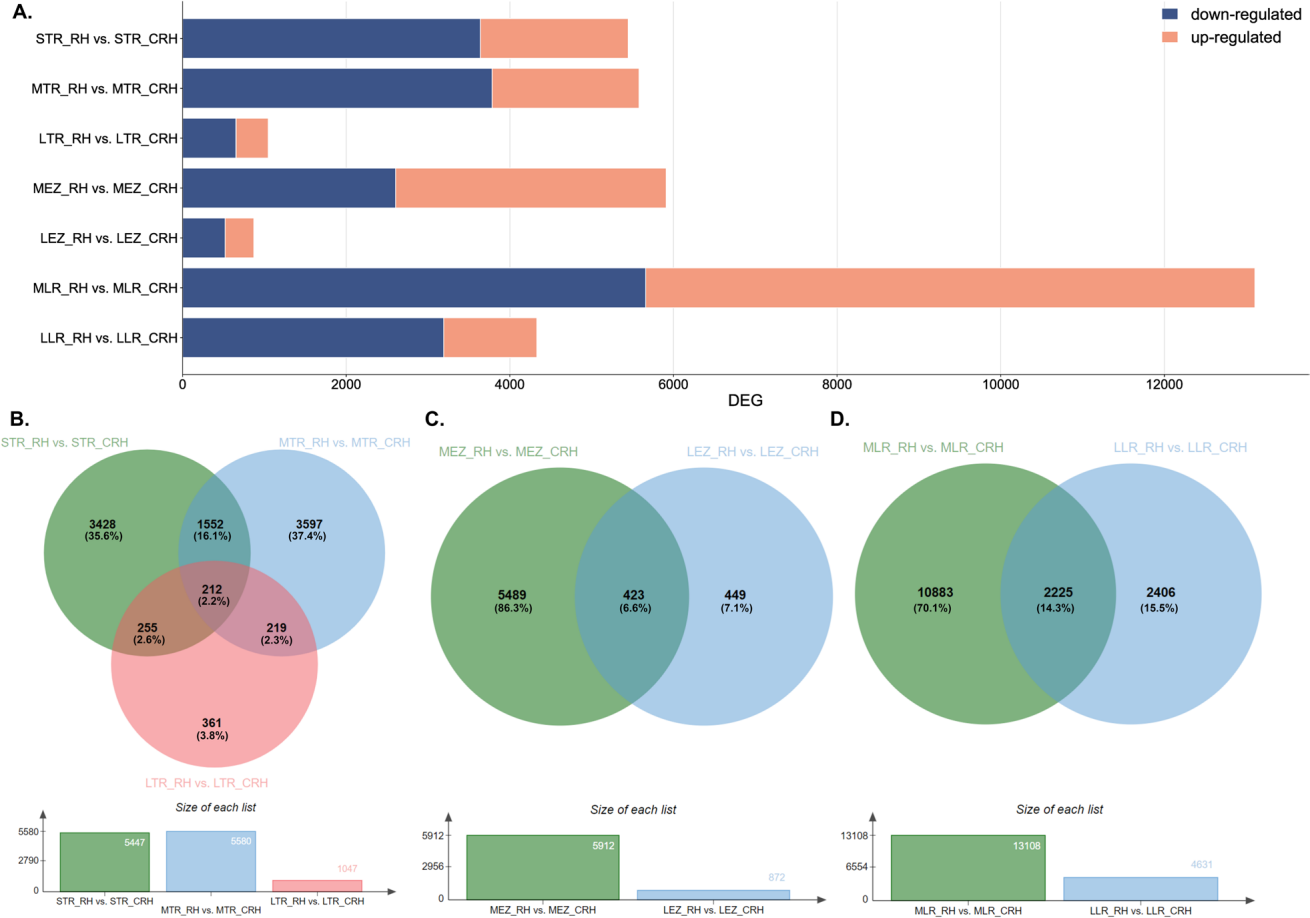
**A) **The number of differentially expressed genes (DEGs) in roots between rhizotron and transplanted comparisons. Venn diagram depicting DEGs identified between **B)** short, medium, and long tips of taproot; **C)** elongation zone from medium and long taproots; **D)** and lateral roots harvested from medium or long taproots based on Bardou et al. (2014) [29]. Annotations with log2FC > 1.5 were used for the analysis of DEGs

To investigate the potential biological processes involved in and regulating taproot elongation after its earlier inhibition in containers, we harvested regrowing taproots from containerized seedlings that were transplanted to rhizotrons the following spring. A comparison between rhizotron-grown and transplanted roots revealed a total of 36,297 differentially expressed genes (DEGs), with 20,052 showing decreased expression and 16,245 being up-regulated in the roots of rhizotron seedlings (Fig. 5A). The number of DEGs that are the same and unique for each comparison is shown in the Venn diagram (Fig. 5B-D). Among the plant cultivation system 212 DEGs exhibited variable expression in meristematic zone of the medium taproot. However, in the elongation zone, this number was almost doubled to 423 (Fig. 5C). This may suggest that cultivation system potentially affected the variations in these genes expression in meristematic as well as in elongation zones. Furthermore, the analysis revealed a significantly higher number of DEGs when comparing gene expression between medium-length roots than between other length categories. This was true regardless of whether the meristematic zone, elongation zone or lateral roots were analyzed. The number of specific DEGs in medium-length roots significantly exceeded the number of DEGs in short or long taproots (Fig. 5B-D).

**Fig. 6:**
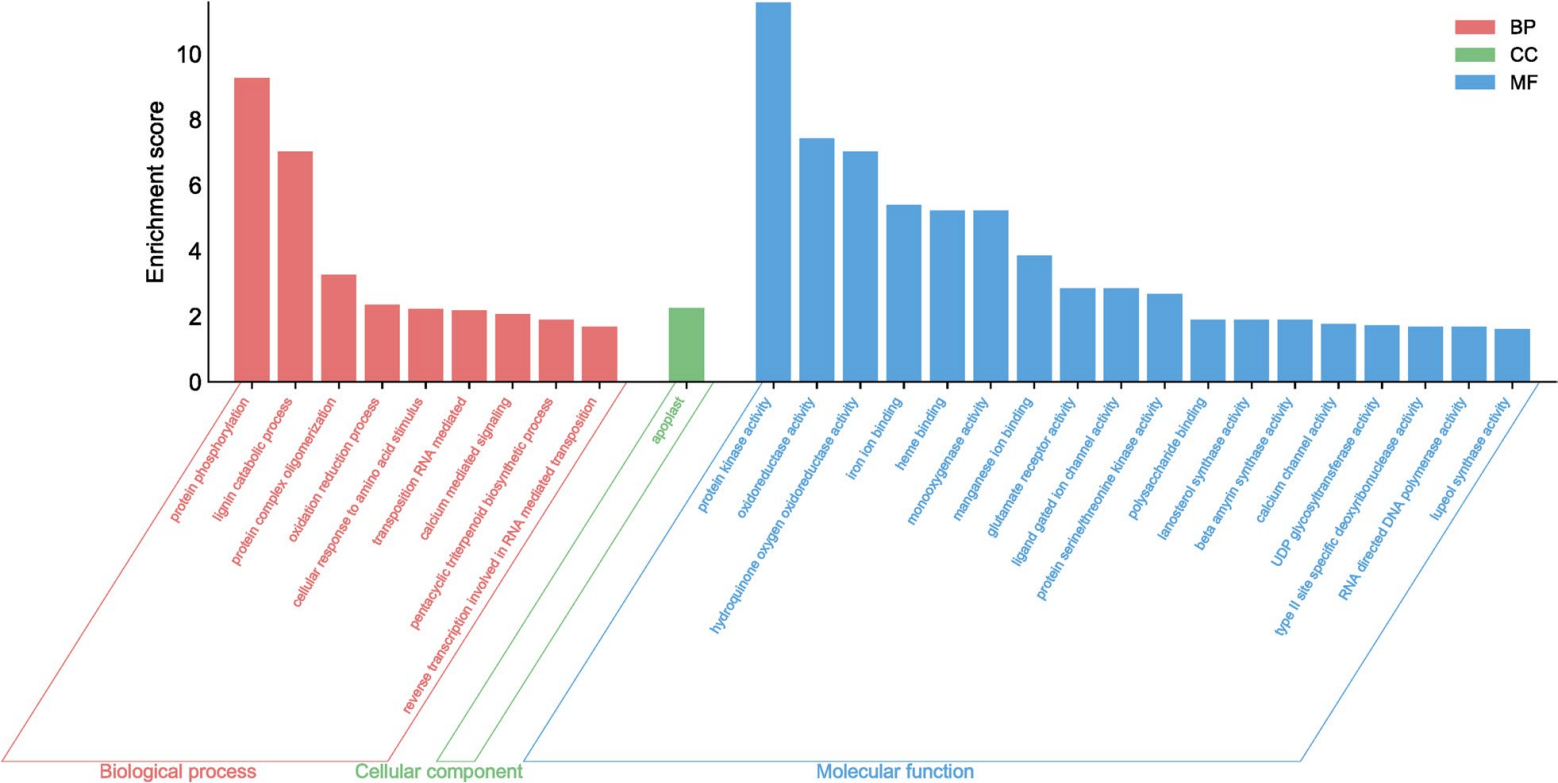
The GO enrichment analysis for the meristematic and elongation zones of taproots of different lengths, and the meristem zone in lateral roots between rhizotron and transplanted comparisons. The enrichment score is represented as -log10(FDR). Enriched GO terms were selected based on an enrichment score < 1.5

By investigating the activation of specific processes within the roots of transplanted seedlings compared to rhizotron-grown seedlings, we found that differentially expressed genes (DEGs) were notably associated with protein phosphorylation, lignin catabolic process, apoplast, and protein kinase activity (Fig. 6).

**Fig. 7:**
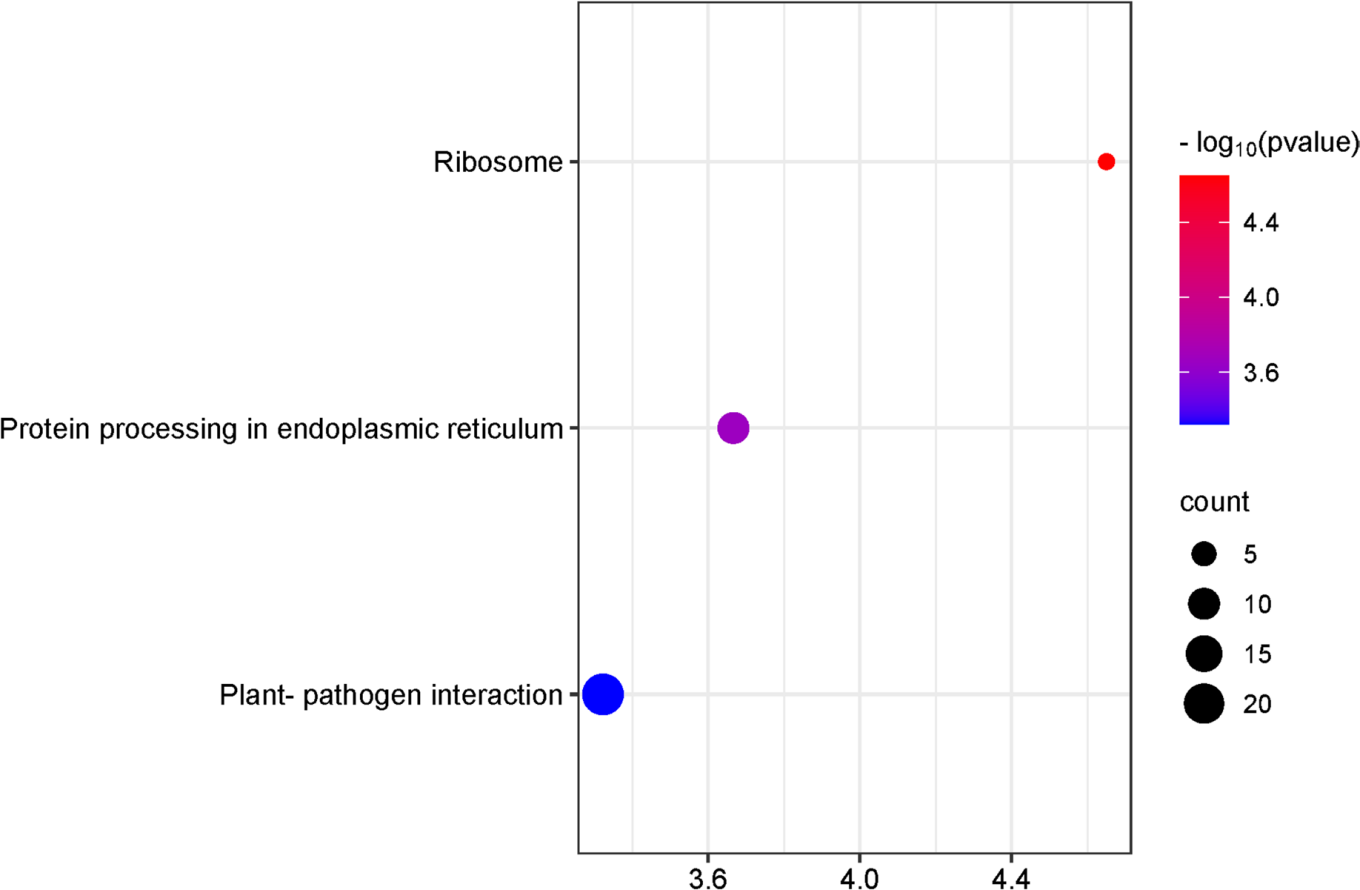
KEGG enrichment analysis of differentially expressed genes (DEGs) in taproot and lateral roots comparing rhizotron and transplanted cultivation systems. The degree of significance of the enrichment of DEGs in a pathway is indicated by -log10 (*p*-value). Circle size represents the number of genes. The color gradient from blue to red indicates low to high enrichment significance [30]

In contrast, we found only 3 significantly enriched KEGG pathways, and the highest expression was associated with protein biosynthesis. The top eight enriched pathways are illustrated in Fig. 7. We selected only those pathways with an FDR < 1.5.

#### Gene expression changes within roots between in container and transplanted comparison

**Fig. 8:**
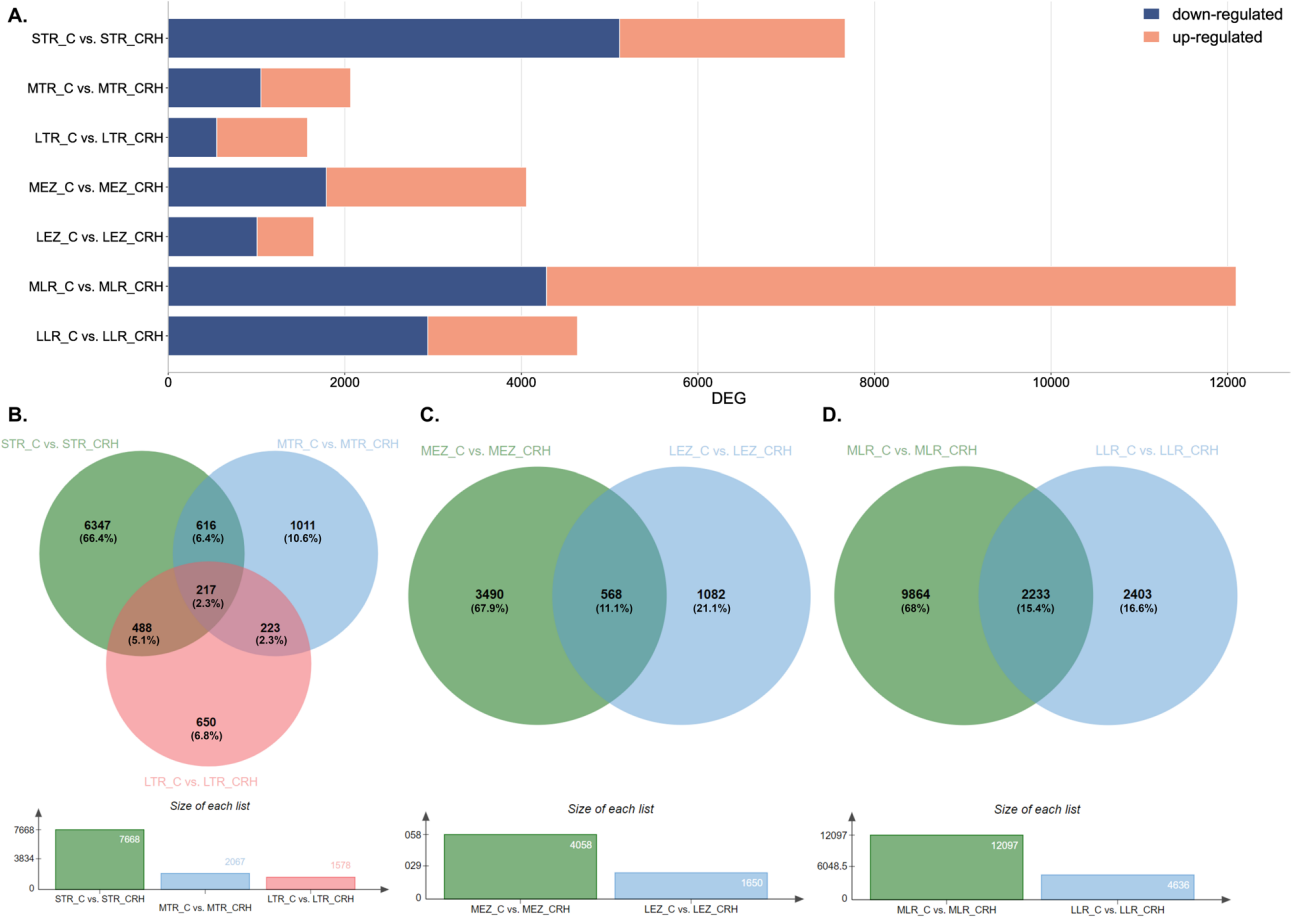
**A)** The number of differentially expressed genes (DEGs) in roots within the container and transplanted comparison. Venn diagram of DEGs identified between **B)** short, medium, and long tips of taproot; **C)** elongation zone from medium and long taproot; **D)** and lateral roots harvested from medium and long length taproots based on Bardou et al. (2014). Annotations with log2FC > 1.5 were used for the analysis of DEGs

We also investigated whether the removal of the growth-inhibiting factor in containers, after transplanting seedlings to the rhizotrons, had a distinct effect on gene expression patterns compared to those observed during taproot elongation in containers. The analysis of differentially expressed genes (DEGs) in oak roots growing in both container and transplanted systems revealed a total of 33,754 DEGs, with 16,735 down-regulated and 17,019 up-regulated in roots growing in containers. Notably, the new roots displayed a higher number of up-regulated genes than down-regulated ones in short taproot (Fig. 8A). This suggests that expression changes occurred after transplantation of seedlings from containers to the rhizotrons and influenced the expression pattern, which differed between taproots elongating in containers and new roots produced after transplantation to rhizotrons. The highest number of DEGs was observed in lateral roots harvested from taproots of medium length, while this number was reduced in long taproots. A similar pattern was observed in the elongation zone of the taproot. The number of DEGs unique for each comparison is shown in the Venn diagram shown in Fig. 8B-D. In the case of the long taproot meristematic zone, 217 DEGs exhibited variable expression depending on the plant cultivation system (Fig. 8B). In the elongation zone, however, this number was more than doubled to 568 (Fig. 5C). This may indicate that not only the different cultivation system may have influenced the variation in expression of these genes, but also the root zone.

Functional analysis comparing container-grown and transplanted roots showed that differentially expressed genes (DEGs) were involved in processes related to secondary metabolites, such as lignin catabolic processes and suberin biosynthesis. Additionally, the analysis revealed processes related to the extracellular region, cell wall, and monooxygenase activity, which are consistent with the intensification of root formation (Fig. 8).

**Fig. 9:**
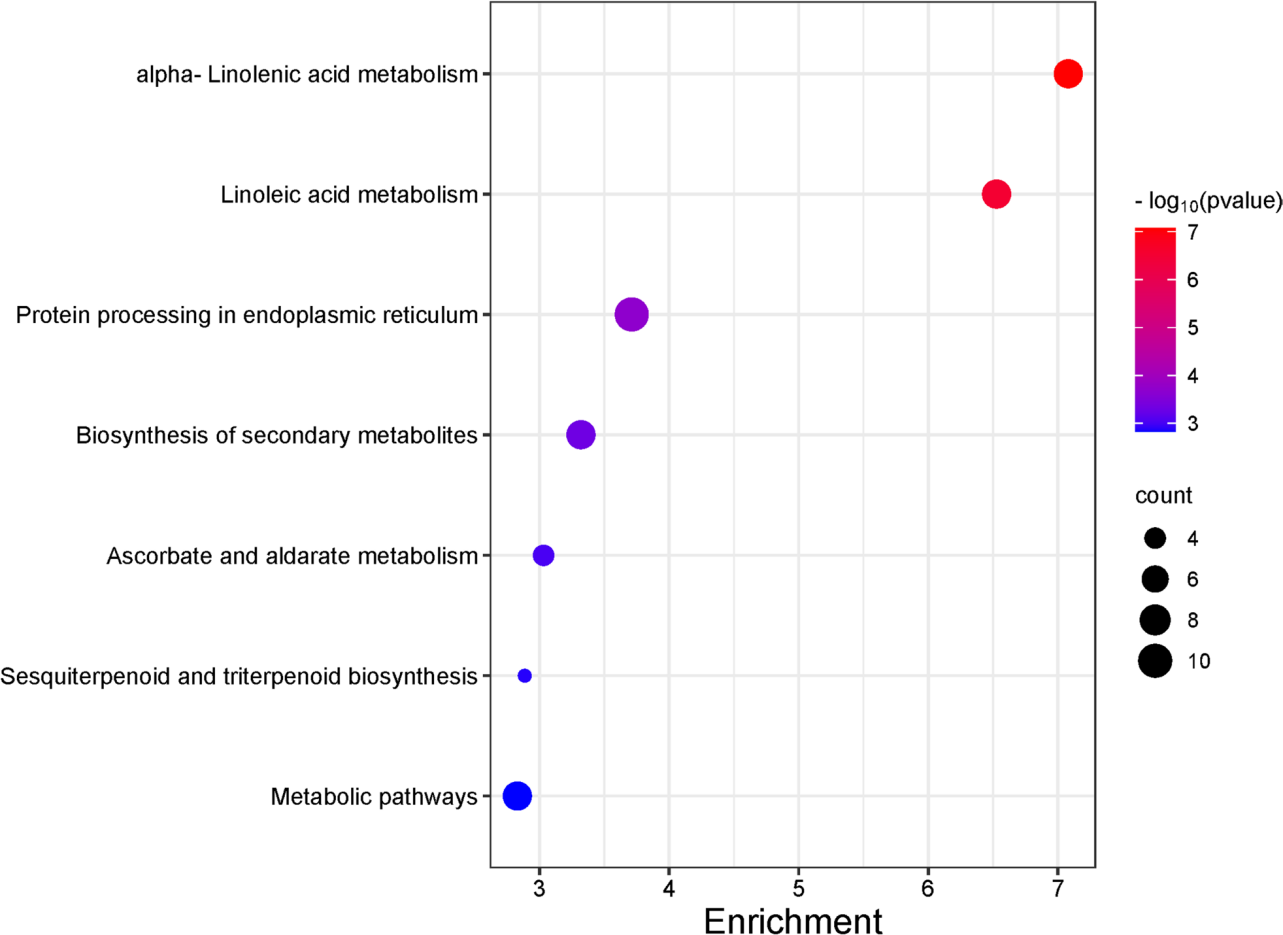
KEGG enrichment analysis of DEGs in taproot and lateral roots within container and transplanted cultivation systems. The degree of significance of the enrichment of differentially expressed genes (DEGs) in a pathway is indicated by -log10 (*p*-value). Circle size represents the number of genes. The color gradient from blue to red indicates the level of enrichment significance, from low to high [30]

In this study, KEGG analysis revealed 7 significantly enriched pathways. The top eight enriched pathways were illustrated in Fig. 9, and many of them were related to linolenic acid metabolism and peptide synthesis. Among the identified KEGG enrichment pathways, we focused on those with an FDR < 1.5.

### Hormonal analysis

To investigate how cultivation systems influence hormone production during the elongation of taproots and lateral roots, we quantified 17 different endogenous phytohormones in various root zones at different stages of growth under different cultivation conditions.

**Fig. 10:**
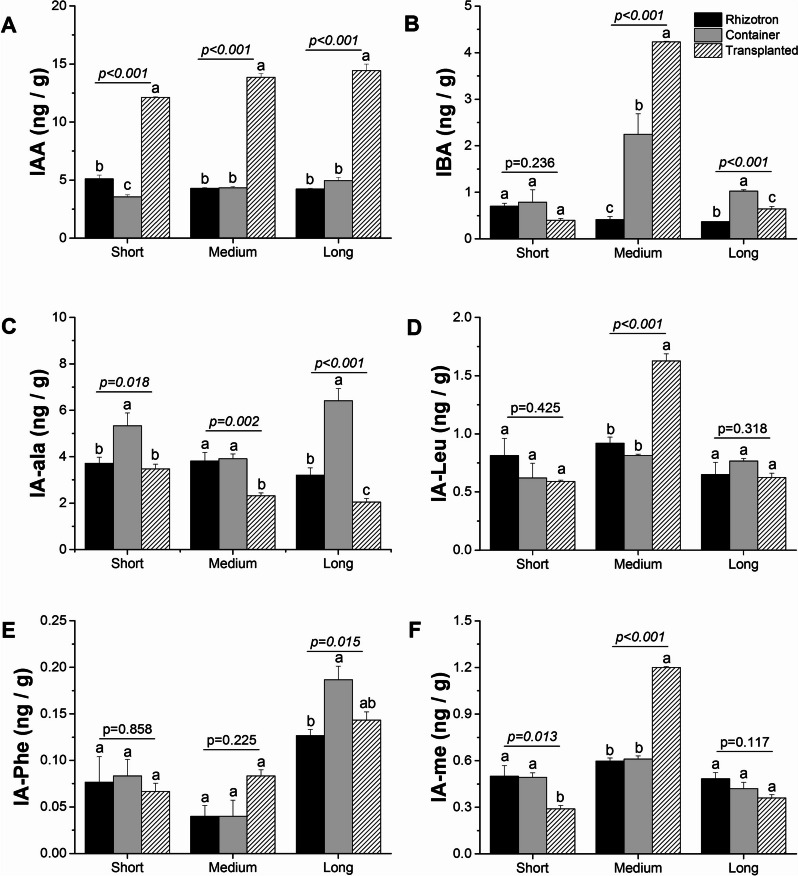
The impact of cultivation systems: rhizotron (black color), container (grey color), and transplanted (hashed lines) on the concentration of IAA (**A**), IBA (**B**), IA-Ala (**C**), IA-Leu (**D**), IA-Phe (**E**), and IA-Me (**F**) in the meristematic zone of short, medium, and long taproots of *Q. robur* seedlings. Each data point represents the mean hormone values for each root length class in each cultivation system, incorporating multiple individual roots from each cultivation system. Hormone concentration values were log10-transformed prior to statistical analysis, but the figures present non-transformed data. The significance of variation between cultivation systems within length classes (short, medium, and long) results from an analysis of variance (ANOVA), as indicated for each length class panel. Different lower-case letters indicate significantly different means among different cultivation systems within a given length class at α = 0.05, according to Tukey’s test. Error bars represent the standard error

For the analysis of endogenous auxin levels in root cells, we measured the concentrations of IAA (indole-3-acetic acid) and its conjugates, including indole-3-acetyl-L-alanine (IA-Ala), indole-3-acetyl-L-leucine (IA-Leu), indole-3-acetyl-L-phenylalanine (IA-Phe) and indole-3-acetyl-L-methionine (IA-Me), and second active form, indole-3-butyric acid (IBA). The concentration of IAA showed a substantial increase in both the meristematic and elongation zones of the transplanted taproots at each stage of elongation. In fact, its concentration was twofold or even more than threefold higher in the transplanted taproots compared to those of the rhizotron or container-grown seedlings (Fig. 10, Fig. S2). Additionally, in lateral roots harvested from medium-length taproots of transplanted seedlings, IAA and its conjugates exhibited significantly higher concentrations compared to those in the rhizotron and container-grown seedlings (Fig. S6).

**Fig. 11:**
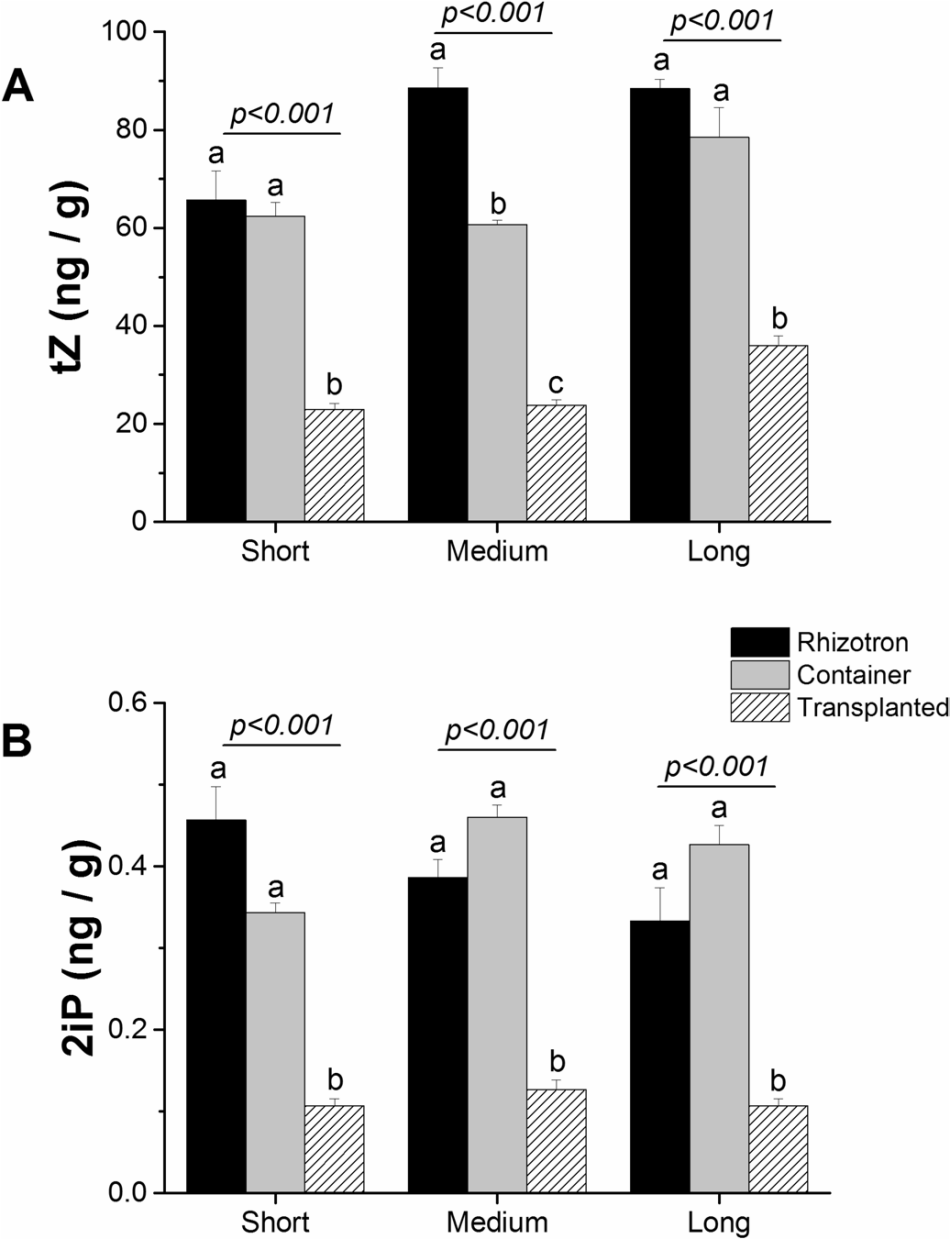
The impact of cultivation systems: rhizotron (black color), container (grey color), and transplanted (hashed lines) on tZ (**A**) and 2iP (**B**) concentrations in the meristematic zone of short, medium, and long taproots of *Q. robur* seedlings. Each point represents the mean hormone values for each root length class in each cultivation system. The data points incorporate multiple individual roots from each cultivation system. Hormone concentration values were log10-transformed before statistical analysis, but figures present non-transformed data. The significance of variation between cultivation systems within length classes, i.e., short, medium, and long, results from an analysis of variance (ANOVA), and the results are presented for each length class panel. Different lowercase letters indicate significantly different means among different cultivation systems within a given length class at α = 0.05 according to Tukey’s test. Error bars represent the standard error

Another hormone we investigated was cytokinins, including trans-Zeatin (tZ) and N6-(2-Isopentenyl)adenine (2iP). Generally, we observed higher levels of endogenous tZ compared to 2iP, across different cultivation systems and root types. Additionally, both hormones exhibited higher concentrations within taproots of rhizotron and container seedlings compared to transplanted seedlings. Notably, there was a visible trend of higher tZ concentration in the medium taproot meristematic zone of rhizotron seedlings compared to container seedlings (Fig. 11). Our analysis indicated no statistically significant differences between the analyzed variants for 2iP in different cultivation systems. However, tZ concentration increased slightly as taproots of rhizotron, container and transplanted seedlings elongated, both in the meristematic and elongation zones of taproot (Fig. 11A, Fig. S2A). Regarding lateral roots, the concentration of both cytokinins was highest when harvested from long-length taproots of transplanted seedlings (Fig. S7). However, a reverse trend was recorded within lateral roots harvested from long taproots.

**Fig. 12:**
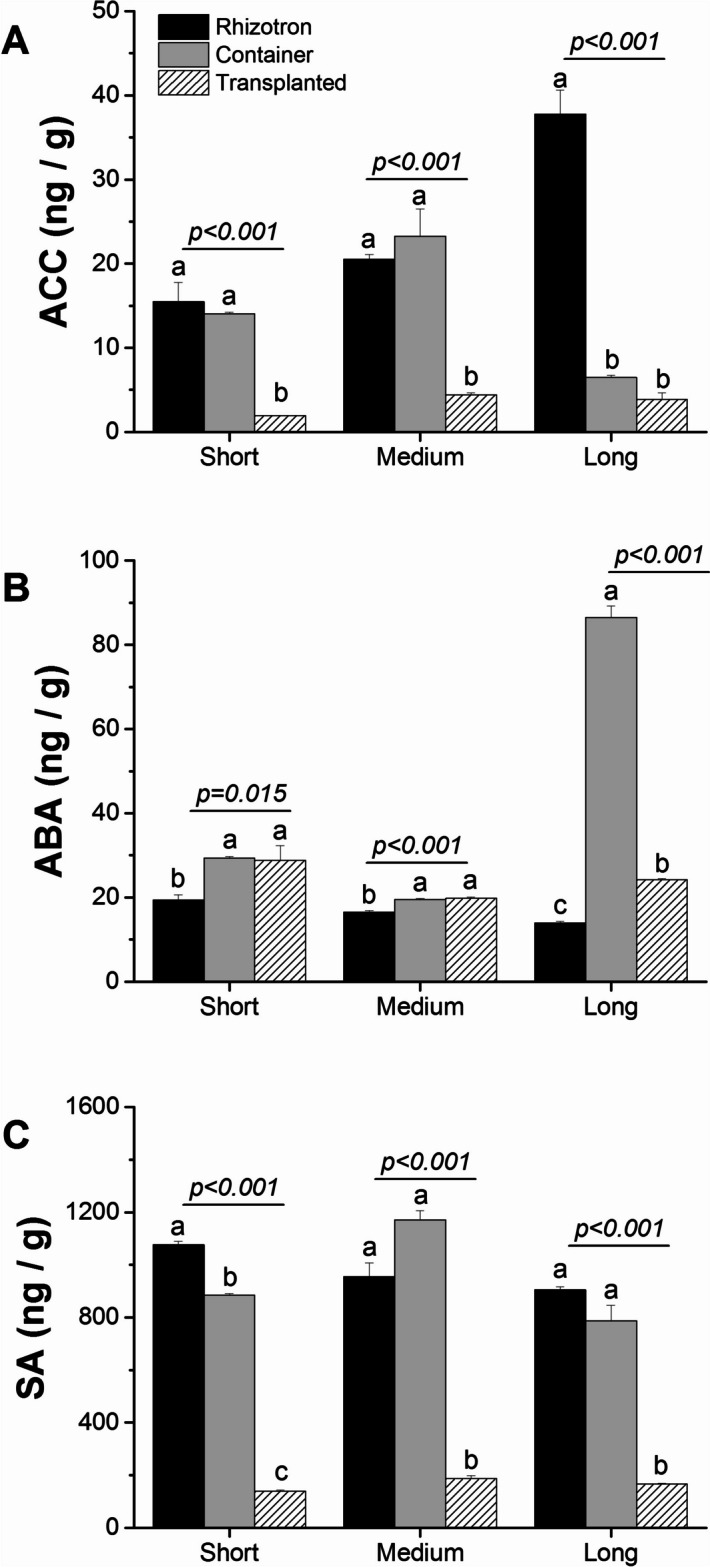
The effect of cultivation systems: rhizotron (black), container (grey), and transplanted (hatched) on ACC (**A**), ABA (**B**), and SA (**C**) concentration in the meristematic zone of short, medium, and long taproots of *Q. robur* seedlings. Each point represents the mean hormone values for each root length class in each cultivation system. The hormone concentration values were log10-transformed before statistical analysis, but figures present non-transformed data. The significance of variation between cultivation systems within length classes (short, medium, and long) results from an analysis of variance (ANOVA) and is given for each length class panel. Different lowercase letters indicate significantly different means among different cultivation systems within a given length class at α = 0.05 according to Tukey's test. Error bars represent the standard error

To understand the interplay between root elongation and ethylene concentration, as well as how different cultivation systems modulate its level, we measured the presence of 1-aminocyclopropane-1-carboxylic acid (ACC), which is the precursor of ethylene, in two zones of taproots and lateral roots. During taproot elongation in the rhizotron, we observed a concomitant increase in ACC in both the meristematic zone (Fig. 12A) and the elongation zone (Fig. S3A) of seedlings. Notably, the elongation zone of taproots growing in the rhizotron showed much higher levels of ACC. In contrast, in the container system, ACC levels peaked when the roots were medium and decreased in long taproots. Additionally, the cultivation system had an impact on ACC levels, with significantly lower concentrations of ACC in taproots growing in the transplanted system. These roots exhibited only marginal changes in all three length classes, compared to roots growing in the container and in the rhizotron. Moreover, the emergence of lateral roots from medium or long taproots had a strong effect on ACC levels, with its concentration increasing in lateral roots harvested from long taproots in containers and transplanted seedlings, while ACC concentration changed only minimally between lateral roots in the rhizotron system (Fig. S8A).

Subsequently, we determined the level of endogenous abscisic acid (ABA) in oak roots, which is known as a stress hormone but also is engaged in root growth regulation. The highest levels of ABA were found in roots growing in the container system at all stages of development in both the meristematic zone (Fig. 12B) and the elongation zone (Fig. S3B). Another hormone, salicylic acid (SA), also showed significantly higher levels in seedlings growing in the container and rhizotron in every root zone and at every stage of elongation (Fig. 12C, Fig. S3C, Fig. S8C).

**Fig. 13:**
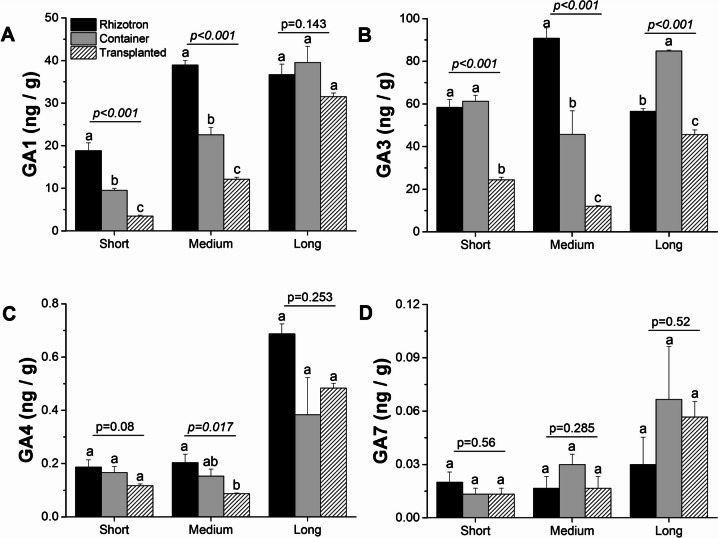
The effect of cultivation systems: rhizotron (black), container (grey), and transplanted (hatched) on GA1 (**A**), GA3 (**B**), GA4 (**C**), and GA7 (**D**) concentrations in the meristematic zone of short, medium, and long taproots of *Q. robur* seedlings. Each point represents the mean hormone values for each root length class in each cultivation system. The hormone concentration values were log10-transformed before statistical analysis, but figures present non-transformed data. The significance of variation between cultivation systems within length classes (short, medium, and long) results from an analysis of variance (ANOVA) and is given for each length class panel. Different lowercase letters indicate significantly different means among different cultivation systems within a given length class at α = 0.05 according to Tukey's test. Error bars represent the standard error

We also examined the levels of four gibberellins: GA3, GA1, GA7, and GA4. During the elongation of short taproots in all analyzed cultivation systems, GA4 and GA7 concentrations increased, irrespective of the root zone (Fig. 13C-D), except GA7 in the elongation zone (Fig. S4D). There were minor differences in GA4 and GA7 concentrations between the cultivation systems. On the other hand, GA1 and GA3 concentrations varied more across cultivation systems, but their concentration increased during taproot elongation in the meristematic zone of container and transplanted seedlings (Fig. 13). The same trend was observed for GA1 and GA3 in the elongation zone of container seedling taproots (Fig. S4A-B). The length of the taproot was also related to the gibberellins concentration within each cultivation system, where the meristematic zone of short and medium taproots of rhizotron and container seedlings had higher concentrations of gibberellins compared to transplanted seedlings. Conversely, the elongation zone of medium taproots displayed the highest concentration of gibberellins. Within lateral roots, the highest concentration of GA7 was visible in the rhizotron system, regardless of which taproot length point they were harvested (Fig. S9D). Transplanted seedlings produced lateral roots that generally had the lowest concentration of all gibberellin classes in medium and long taproots.

**Fig. 14:**
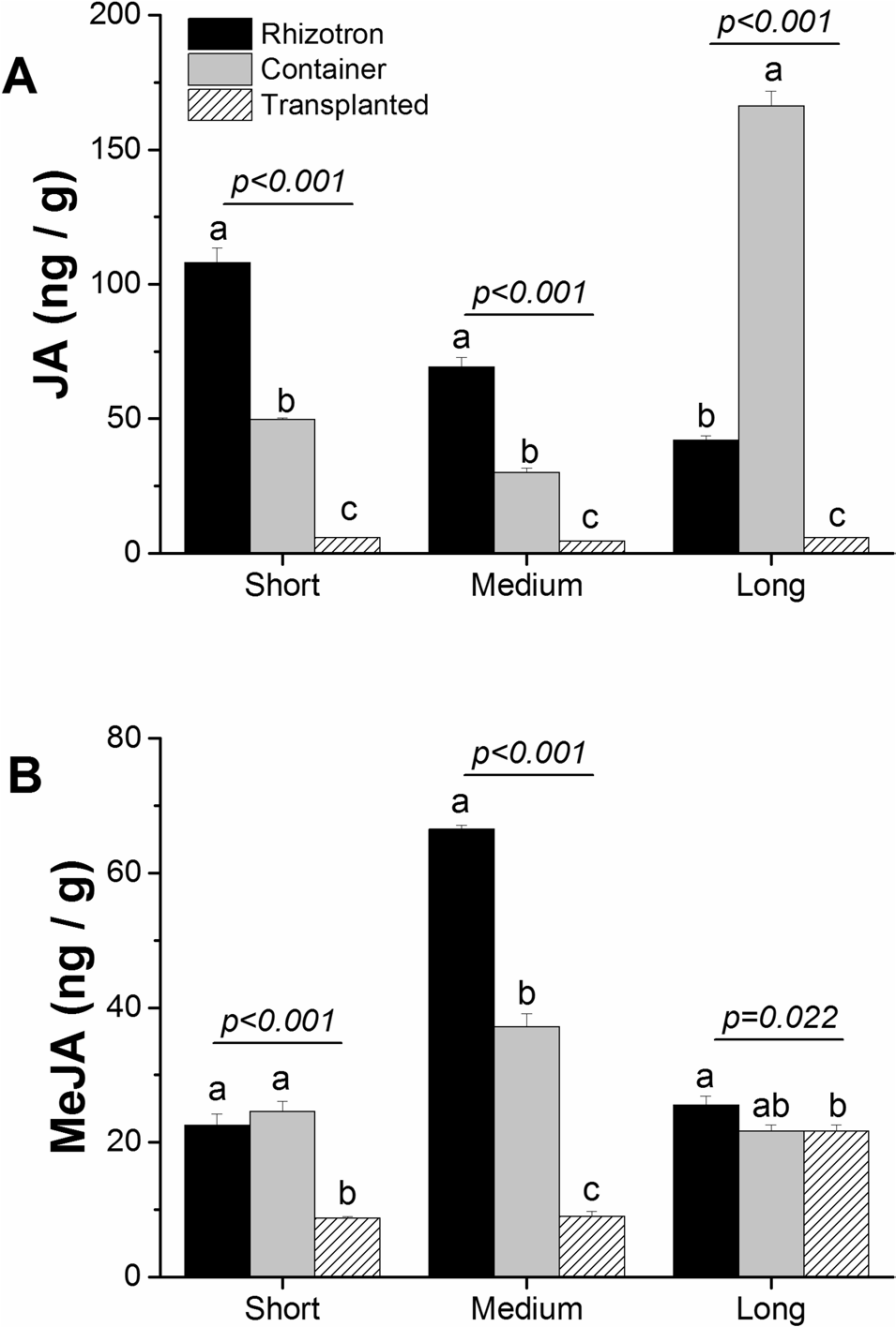
The impact of cultivation systems (rhizotron—black color, container—grey color, and transplanted—hashed) on JA (**A**) and MeJA (**B**) concentrations in the meristematic zone of short, medium, and long taproots of *Q. robur* seedlings. Each data point represents the mean hormone values for each root length class in each cultivation system, incorporating multiple individual roots from each cultivation system. Hormone concentration values were log_10_-transformed before statistical analysis, but figures present non-transformed data. Significance of variation between cultivation systems within length classes (i.e., short, medium, and long) results from an analysis of variance (ANOVA) and is indicated for each length class panel. Different lowercase letters indicate significantly different means among different cultivation systems within a given length class at α = 0.05, as determined by Tukey's test. Error bars represent the standard error

In addition, we examined the levels of jasmonic acid (JA) and its derivative methyl jasmonate (MeJA). The analysis revealed a significant decrease in both JA and MeJA in roots growing in the transplanted system compared to roots growing in the container and in the rhizotron (Fig. 14, Fig. S5, Fig. S10).

## Discussion

Root elongation in trees is regulated by a complex interplay of internal and external factors, mediated through intricate signaling pathways [13, 14, 31, 32]. Understanding the integration of these internal and external signals in modulating root growth is of particular significance for tree cultivation. Despite substantial research, many essential questions remain unanswered concerning the specific signals and signaling pathways that govern taproot growth. Therefore, our study aims to illustrate and confirm the general pattern of taproot growth and lateral root emergence. However, we have also observed that gene expression and hormone production within the root tip of the taproots play a central role in contributing to growth organization in different cultivation systems and act as regulators of lateral root formation. Through differential gene expression analysis and functional annotations (GO and KEGG), we have identified factors involved in supporting taproot growth, as well as those halting its elongation and delaying lateral root formation. This evidence indicates that signaling arising from taproot tips likely reflects the developmental requirements of the taproot system for different hormones. To the best of our knowledge, this study represents the first report investigating gene expression changes between distinct cultivation systems, encompassing different taproot tissues (meristematic and elongation zones) and various root types (taproot and lateral root) in trees. These findings contribute valuable insights into the regulatory mechanisms of root growth in trees and can have significant implications for tree cultivation and management practices.

### Profiling of differentially expressed genes in roots growing in different cultivation systems

Our transcriptome analysis revealed that as the taproot grows, the number of differentially expressed genes (DEGs) increases when comparing container-grown and rhizotron-grown seedlings (Fig. 2). Interestingly, we observed an increased number of down-regulated DEGs during taproot elongation in the meristematic and elongation zones of rhizotron-grown seedlings, suggesting induced expression of genes in container-grown seedlings. This response may be attributed to container conditions, where taproots sense air at the bottom of the container, resulting in a large increase in DEG expression, likely reflecting signals that hamper root growth. In contrast, undisturbed taproot elongation of rhizotron seedlings is not negatively affected. On the other hand, a significant decrease in the number of genes involved in root growth can be observed in long roots, both in the meristematic and elongation zones, when comparing rhizotron-grown and transplanted seedlings (Fig. 5). The enhanced DEGs expression in transplanted and rhizotron-grown seedlings could be an adaptive response involved in the modulation of root growth extension after germination [33]. Since the ability of trees ability to access water in deeper soil layers is crucial to minimize water stress during periods of drought [5, 6, 10], this potential might enable rapid adjustment of taproot growth to regulate water absorption under unfavorable conditions, whereas under unaltered growth, fewer genes are involved in root elongation. When comparing root growth between container and transplanted seedlings, the results showed that the ability of trees to restart growth of taproots relies on the enhanced expression of genes. Observed down-regulation in container seedlings may have affected their taproot growth cessation earlier, possibly shortly after germination or when reaching a medium length. It appears that sequential events at the level of gene expression are related to taproot growth within container seedlings, with both the apical meristem and elongation zone responding to growth conditions. Conversely, the up-regulation of a series of genes also defines the recovery potential of transplanted seedlings as their replanting from containers to rhizotrons induces rapid changes in gene expression, which gradually decrease with root elongation, reaching expression levels similar to seedlings growing in rhizotrons, especially when roots are longer. This decrease is coincidental with the observed duration of elongation, as the initial phase after germination exhibits the highest growth rate with high cell proliferation [34]. Therefore, it can be inferred that container cultivation of pedunculate oak induces gene expression changes that may influence the further growth of these plants, after transplantation into natural conditions. These findings shed light on the molecular mechanisms underlying root growth regulation in different cultivation systems and provide valuable insights into the adaptability and recovery potential of oak seedlings under changing environmental conditions.

The functional GO analysis revealed that genes associated with "cellular response to amino acid stimulus" exhibited the highest activity in roots growing in rhizotrons compared to containers (Fig. 3). This suggests that transcriptional factors regulating changes in cell state or activity in response to an amino acid stimulus may play a crucial role in promoting taproot rooting in rhizotrons. Considering that the roots in rhizotrons and containers are relatively young (shortly after germination), the enhanced expression of glutamate receptor family genes (*GLR27*, *GLR25*, *GLT24*) may promote taproot elongation, as their expression was reduced to a higher degree in container seedlings. Furthermore, in rhizotron taproots, there was an increase in the expression of genes associated with ion processing, such as "calcium ion transport," "calcium-mediated signaling," "calcium channel activity," and "glutamate receptor activity." These genes are involved in calcium signaling [35], suggesting that this rise in expression is necessary for maintaining continuous root growth. Calcium is involved in processes such as regulating primary root growth through auxin signaling, modulating primary root growth through cytokinin signaling, promoting primary root elongation by interfering with brassinosteroid signaling, and being involved in abscisic acid-inhibited root growth through ROS signal transduction or influencing ethylene biosynthesis plays a significant role in growth and development [36]. Additionally, calcium facilitates primary root growth by regulating cell wall reformation and is also involved in root development in the absence of sucrose [37]. It also influences key regulators of root growth, *PLETHORA1* and *PLETHORA2*, and positively modulates root meristem size and promotes primary root growth through interaction with the Arabidopsis glutamate receptor-like protein AtGLR3.6 [37]. These results confirm that root growth regulation is determined early, and environmental manipulation affecting calcium-induced gene expression may provide an opportunity to promote specific gene expression-hormone production and thereby affect taproot growth of container seedlings before they reach the bottom of the container and die. The suppressed expression of calcium-related genes in container seedlings supports our assumption that calcium may be a key factor promoting root elongation and growth.

In the comparison between roots growing in the rhizotron and transplanted roots, we observed an enrichment of genes associated with developmental processes such as "protein phosphorylation" and "protein kinase activity." Protein phosphorylation plays a crucial role in metabolism and signaling pathways. Additionally, genes related to "lignin catabolic process" were also observed (Fig. 6). Lignin in cells provides an extracellular barrier to solutes and water and plays a key role in maintaining nutrient homeostasis [38]. The increased activity of genes associated with the "apoplast" may indicate enhanced water transport for the well-developed shoot of transplanted seedlings that is supported by the enhanced expression of *AGT1*, *GL17*, *GP1*, and *EXL7*. Interestingly, our results indicate that genes expressed in the taproot of rhizotron and transplanted seedlings may have similar functions, with growth-promoting processes possibly even higher in the restored taproot of transplanted seedlings (Fig. 6), confirming the existence of recovery potential enabling growth taproots growth that were previously hampered due to environmental factors or cultivation conditions in containers. Especially that in transplanted seedlings, when comparing to containers, we found the enhanced activity of genes associated with lignification and suberization that regulate water and nutrient absorption and enhance root mechanical resistance [39, 40].

**Fig. 15:**
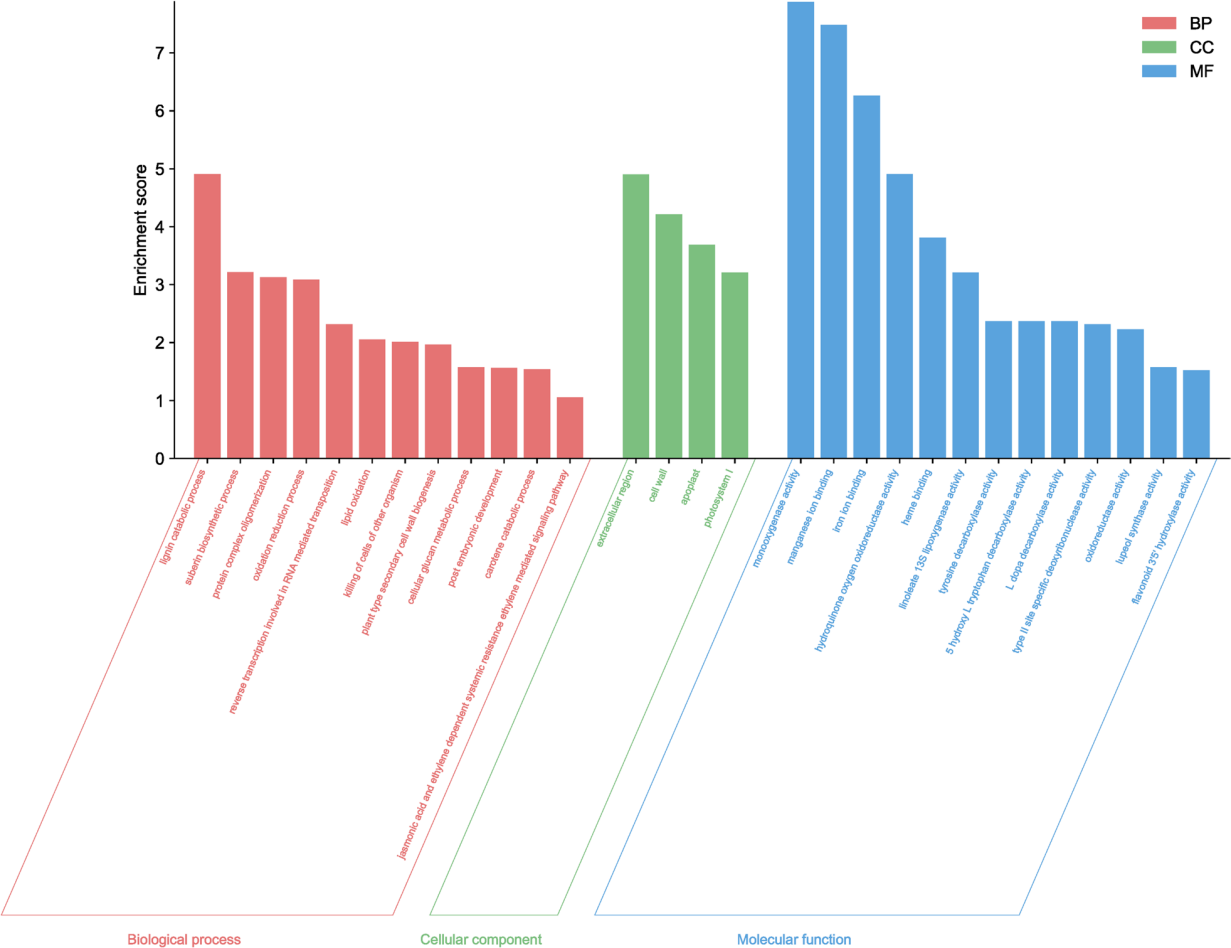
GO enrichment analysis of differentially expressed genes (DEGs) in the meristematic and elongation zones of taproots of different lengths and the meristem zone in lateral roots, comparing container-grown and transplanted roots. The enrichment score is represented as-log10(FDR). Enriched GO terms were selected based on an enrichment score < 1.5

Other genes related to "protein complex oligomerization," "extracellular region," "lipid oxidation," and "linoleate 13S lipoxygenase activity" may indicate the activation of intense developmental processes and tissue specialization, possibly leading to an increased number of genes associated with the "lignin catabolic process" (Fig. 8). The increased metabolic pathway observed in the KEGG analysis related to fatty acid production (Fig. 15) may indicate enhanced production of jasmonic acid, which participates in the response to pathogen attacks but also may promote root development, acting as a modulator of auxin homeostasis [41, 42]. It appears that transplanted seedlings promote taproot growth after planting in the rhizotron, whereas taproots of seedlings growing in containers promoting vigorous lateral root growth what may be associated with limited space for taproot growth and its inhibition before the root reaches the bottom of the container. However, confirmation of this thesis requires additional research and verification.

### Analysis of plant hormones in roots

The molecules that elicit and regulate root growth and development in plants are known as plant hormones, and they can simultaneously induce root growth initiation as well as root growth inhibition. For example in Arabidopsis, auxin has been shown to act as either a positive or negative regulator of primary root growth, depending on its concentration. High concentrations of auxin (~ 10^–6^ M) impede primary root elongation, while very low concentrations (~ 10^–8^ M) promote root elongation, and the effect may be tissue and zone-specific [16, 43]. However, there is limited research pertaining to the impact of hormonal interactions on taproot and lateral root elongation under different nursery cultivation systems of forest trees. Hence, it is crucial to examine hormone levels at different plant growth stages and measure the concentrations of other hormones when analyzing plant hormone effects, especially in long-lived trees. Analyzing different forms of hormones, we revealed that IAA is the dominant form of auxin, and its concentration differs depending on the cultivation system. Roots grown in the transplanted system exhibited a different pattern of auxin levels compared to those grown in containers and rhizotrons, both in the meristematic and elongation zones of the taproot. As the taproot elongated, the level of IAA also increased in taproots of seedlings grown in transplanted system, contrasting with seedlings grown in the rhizotron and container in the meristematic zone. However, a decrease in IAA as taproot elongation occurred was observed in lateral roots of transplanted seedlings. When transplanting seedlings from containers to rhizotrons, we observed the activation of genes involved in auxin biosynthesis in both taproot and lateral roots, as revealed by the analysis of differentially expressed genes (DEGs), in which expression levels of auxin-encoding genes were higher in transplanted seedlings than in rhizotron seedlings (Table S2). Hence, high auxin concentration is required for taproot elongation of transplanted seedlings, while high auxin concentration in taproot tips may operate downstream to regulate lateral root growth by direct interaction with increased levels of cytokinin as indicated for Arabidopsis by Aloni et al. [18], and manifests apical dominance of taproots, which promotes the growth of the primary root while inhibiting lateral root initiation, allowing deeper soil exploration over longer distances [18, 44]. In Arabidopsis, cytokinin can inhibit lateral root growth by reducing the expression of *PIN* genes, which encode auxin efflux carriers in lateral root founder cells [45]. Consequently, cytokinin regulates root architecture by balancing the promoting role of auxin in lateral root development in organisms with a long main root [18, 46]. Our findings of lower cytokinin concentration in the meristematic and elongation zones within taproots and high auxin concentration in lateral roots harvested from medium taproots of transplanted seedlings but higher levels of cytokinin in the meristematic and elongation zones of the taproot observed in rhizotron and container seedlings, support the conclusion that apical dominance within transplanted plants forms later than in latter cultivation systems. The explanation for this contradictory observation is that a lower auxin-to-cytokinin ratio that promote taproot growth in rhizotron but is formed later in transplanted seedlings processes due to time necessary for reestablishment of taproot growth, as indicated by elevated cytokinin concentration in LLR within transplanted seedlings, as the main form of cytokinin, i.e., tZ, slightly increased with root elongation, especially among transplanted seedlings. This, along with the decreasing IAA level, indicates the involvement of an auxin/cytokinin balance in root elongation of transplanted seedlings. The ability to access water from deeper sources is crucial for maintaining tree vitality and services under ongoing and predicted warming. For instance, drought stress can decrease cytokinin synthesis while simultaneously increasing auxin levels, thereby promoting taproot elongation [47].

In addition to the auxin-cytokinin balance, our study also revealed the involvement of gibberellins in promoting taproot growth. This is supported by higher concentrations of dominant gibberellin forms (GA1, GA3) within the meristematic and elongation zone (GA3) of short and medium taproots in both rhizotron and transplanted seedlings. This finding confirms earlier reports of the stimulatory potential of gibberellins on primary root growth [48], especially through their interaction with auxin [49]. Furthermore, the interplay between auxins and gibberellins may modulate taproot elongation in transplanted seedlings. For a visual comparison, please refer to Figure S1 and Figure S4, which show the concentrations of auxins and gibberellins within the elongation zone of transplanted seedlings.

We also observed a concomitant increase in the ethylene precursor ACC in taproots grown in container and rhizotron systems compared with transplanted seedlings. The presence of a high level of ACC in medium taproots of container seedlings, simultaneously with an enhanced presence of auxin in long taproots of the same cultivation system, suggests that the inhibition process must have taken place before root growth cessation. The clue lies in how the pathway of successive hormone involvement is controlled. High concentrations of ethylene can inhibit primary root growth by suppressing cell proliferation in the apical meristem and the elongation zone [20, 50]. Additionally, the observed sharp decrease in ACC levels in long roots of container seedlings (Fig. 12A), along with higher concentrations of IBA and IA-ala, and a slight increase in tZ at the short and medium taproots, seems to impede their elongation before they reach the bottom of the containers. This effect appears to be due to these factors rather than solely the presence of ethylene itself. In the system with undisturbed growth i.e. rhizotron the enhanced concentration of ACC in long taproots of rhizotron seedlings suggests that its high level in itself is not sufficient for hampering root growth, as continuous taproot growth of rhizotron seedlings is supported by enhanced concentrations of tZ and gibberellins, which are necessary and crucial for continuous growth [51], confirming that the manner by which ethylene controls root inhibition patterning in container seedlings dependent on other hormones Thus, based on the given hormone ratio (lower IAA concentration than CK and predicted higher ET concentration than IAA), it can be presumed how the growth of the main root would be inhibited, especially in container seedlings. The abscisic acid (ABA) synthesis, where low concentrations stimulate primary root growth, while high concentrations have inhibitory effects [52]. In this scenario, growth promotion by ABA at low concentrations is independent of ethylene action and only requires auxin signaling and its transport through auxin efflux carriers, whereas inhibitory effects of ABA at high concentrations would be regulated through auxin and elevated concentration of ethylene [52]. The fact that taproots of rhizotron and container seedlings produced a low amount of ABA, but ABA concentration significantly increased in container seedlings, indicates that this hormone may not promote root elongation but may operate as a factor perpetuating growth inhibition induced earlier by ethylene. The high ABA levels observed simultaneously with only slight changes in GA concentrations in the meristematic or elongation zone of taproots within container seedlings confirm that in oaks, the ABA-Gibberellin complex does not act in a regulatory feedback loop inhibiting elongation of taproot cells and growth inhibition is more likely to be due to the effects of ethylene [53]. Also, other hormones such as jasmonic acid (JA) and its methylated form (MeJA), which are enhanced in concentration in the lateral roots of container seedlings (Fig. S10) and show higher expression of genes involved in jasmonate biosynthesis and fatty acid production (KEGG) – serving as a precursor for JA biosynthesis [41], may be directly involved in the inhibition of taproot growth [54], and should promote lateral root growth in container seedlings. However, it should be noted that the influence of hormones on root growth is complex and dependent on multiple factors, such as plant species, developmental stage, environmental conditions, and molecular regulatory factors. Nevertheless, our DEG analysis of genes encoding hormone biosynthesis precursors showed a similar pattern to the plant hormone level analysis (Table S2).

## Conclusion

In summary, our transcriptome analysis provided valuable insights into the gene expression patterns and hormone involvement during taproot growth in different cultivation systems. This container bottom response, triggered by the taproots sensing air in the container, could result in signals that hinder taproot growth in some seedlings. In contrast, rhizotron-grown seedlings exhibited taproot elongation without negative effects on gene expression. Comparing rhizotron-grown and transplanted seedlings, we observed a significant decrease in the number of genes involved in root development in long taproots of rhizotron seedlings. The up-regulation of DEGs in transplanted seedlings is related to taproot growth, indicating a recovery potential for growth in taproots that were hampered when seedlings were previously growing in the container, and the ability of trees to restart growth after transplantation relies on enhanced gene expression, promoting elongation mechanisms This recovery potential may play a crucial role in adjusting taproot growth for water absorption from deeper soil layers under water scarcity. Functional GO analysis revealed that genes associated with "cellular response to amino acid stimulus" exhibited the highest activity in roots growing in rhizotrons, indicating that transcription factors responding to amino acid stimuli promote taproot rooting in this system. Enhanced expression of glutamate receptor family genes in rhizotron taproots suggests their role in taproot elongation. Additionally, genes associated with ion processing and calcium signaling were up-regulated in rhizotron taproots, highlighting their importance in maintaining continuous root growth. Comparing roots growing in containers and transplanted roots, genes involved in the "suberin biosynthesis process" were most active in container seedlings, reflecting root response to stress within container seedlings. Analyzing the hormone profiles, we found that auxin (IAA) concentration differed among the cultivation systems, with higher levels in taproot of transplanted seedlings. Cytokinin and gibberellin levels were higher in rhizotron, thus promoting continuous taproot growth. ABA showed complex patterns, indicating its involvement in taproot growth inhibition of container seedlings. In conclusion, our study demonstrated that transplanting roots from containers to rhizotrons activates a series of molecular reactions that promote taproot growth, while plants grown in containers may restrict taproot growth through a complex hormone network. Understanding these molecular mechanisms can help optimize cultivation practices and promote healthy root growth, especially in the early stages of seedling growth and after transplantation into the field. Further research is required to confirm and expand upon these findings and explore the interactions between multiple endogenous factors and their impact on root development, particularly in species other than model plants.

### Supplementary Information

**Additional file 1: Fig. S1.** The effect of cultivation systems: rhizotron (black color), container (grey color) and transplanted (hacked) on IAA (A), IBA (B), IA-Ala (C), IA-Leu (D), IA-Phe (E), IA-Me (F) concentration in elongation zone of short, medium and long taproots of *Q. robur* seedlings. **Fig. S2.** The effect of cultivation systems: rhizotron (black color), container (grey color) and transplanted (hacked) on tZ (A), 2iP (B) concentration in elongation zone of short, medium and long taproots of *Q. robur* seedlings. **Fig. S3.** The effect of cultivation systems: rhizotron (black color), container (grey color) and transplanted (hacked) on ACC (A), ABA (B), SA (C) concentration in elongation zone of short, medium and long taproots of *Q. robur* seedlings. **Fig. S4.** The effect of cultivation systems: rhizotron (black color), container (grey color) and transplanted (hacked) on GA1 (A), GA3 (B), GA4 (C) GA7 (D) concentration in elongation zone of short, medium and long taproots of *Q. robur* seedlings. **Fig. S5.** The effect of cultivation systems: rhizotron (black color), container (grey color) and transplanted (hacked) on JA (A), MeJA (B) concentration in elongation zone of short, medium and long taproots of *Q. robur* seedlings. **Fig. S6.** The effect of cultivation systems: rhizotron (black color), container (grey color) and transplanted (hacked) on IAA (A), IBA (B), IA-Ala (C), IA-Leu (D), IA-Phe (E), IA-Me (F) concentration in meristematic zone of medium and long lateral roots of *Q. robur* seedlings. **Fig. S7.** The effect of cultivation systems: rhizotron (black color), container (grey color) and transplanted (hacked) on tZ (A), 2iP (B) concentration in meristematic zone of medium and long lateral root of *Q. robur* seedlings. **Fig. S8.** The effect of cultivation systems: rhizotron (black color), container (grey color) and transplanted (hacked) on ACC (A), ABA (B), SA (C) concentration in meristematic zone of medium and long lateral roots of *Q. robur* seedlings. **Fig. S9.** The effect of cultivation systems: rhizotron (black color), container (grey color) and transplanted (hacked) on GA1 (A), GA3 (B), GA4 (C) GA7 (D) concentration in meristematic zone of medium and long lateral roots of *Q. robur* seedlings. **Fig. S10.** The effect of cultivation systems: rhizotron (black color), container (grey color) and transplanted (hacked) on JA (A), MeJA (B) concentration in meristematic zone of medium and long lateral roots of *Q. robur* seedlings.

**Additional file 2: Table S1.** MRM values and positive (+) and negative (-) ionization used in the analysis of phytohormones and deuterated standards.

**Additional file 3: Table S2.** Differential expression patterns of plant hormone biosynthesis related genes in library comparison between roots in different cultivation systems.

## Data Availability

The datasets generated and/or analyzed during the current study are available in the NCBI GEO repository, with accession number GSE181860 (https://www.ncbi.nlm.nih.gov/geo/query/acc.cgi?acc=GSE181860) and OakRootRNADB Database (https://oakrootrnadb.idpan.poznan.pl/). All data generated or analyzed during this study are included in this published article and its supplementary information files.

## Abbreviations

STR_RH: Short taproot, meristematic zone, rhizotron
STR_C: Short taproot, meristematic zone, container
STR_CRH: Short taproot, meristematic zone, transplanted
MTR_RH: Medium taproot, meristematic zone, rhizotron
MTR_C: Medium taproot, meristematic zone, container
MTR_CRH: Medium taproot, meristematic zone, transplanted
LTR_RH: Long taproot, meristematic zone, rhizotron
LTR_C: Long taproot, meristematic zone, container
LTR_CRH: Long taproot, meristematic zone, transplanted
MEZ_RH: Medium taproot, elongation zone, rhizotron
MEZ_C: Medium taproot, elongation zone, container
MEZ_CRH: Medium taproot, elongation zone, transplanted
LEZ_RH: Long taproot, elongation zone, rhizotron
LEZ_C: Long taproot, elongation zone, container
LEZ_CRH: Long taproot, elongation zone, transplanted
MLR_RH: Lateral root from medium taproot, meristematic zone, rhizotron
MLR_C: Lateral root from medium taproot, meristematic zone, container
MLR_CRH: Lateral root from medium taproot, meristematic zone, transplanted
LLR_RH: Lateral root from long taproot, meristematic zone, rhizotron
LLR_C: Lateral root from long taproot, meristematic zone, container
LLR_CRH: Lateral root from long taproot, meristematic zone, transplanted
