# Supplementary material for: Identification of genetics and hormonal factors involved in Quercus robur root growth regulation in different cultivation system
Source: BMC Plant Biol. 2024 Feb 20;24:123. doi: 10.1186/s12870-024-04797-z (PMC10877882; doi:10.1186/s12870-024-04797-z)
Supplement: Supplementary file 1 — Additional file 1: Fig. S1. The effect of cultivation systems: rhizotron (black color), container (grey color) and transplanted (hacked) on IAA (A), IBA (B), IA-Ala (C), IA-Leu (D), IA-Phe (E), IA-Me (F) concentration in elongation zone of short, medium and long taproots of Q. robur seedlings. Fig. S2. The effect of cultivation systems: rhizotron (black color), container (grey color) and transplanted (hacked) on tZ (A), 2iP (B) concentration in elongation zone of short, medium and long taproots of Q. robur seedlings. Fig. S3. The effect of cultivation systems: rhizotron (black color), container (grey color) and transplanted (hacked) on ACC (A), ABA (B), SA (C) concentration in elongation zone of short, medium and long taproots of Q. robur seedlings. Fig. S4. The effect of cultivation systems: rhizotron (black color), container (grey color) and transplanted (hacked) on GA1 (A), GA3 (B), GA4 (C) GA7 (D) concentration in elongation zone of short, medium and long taproots of Q. robur seedlings. Fig. S5. The effect of cultivation systems: rhizotron (black color), container (grey color) and transplanted (hacked) on JA (A), MeJA (B) concentration in elongation zone of short, medium and long taproots of Q. robur seedlings. Fig. S6. The effect of cultivation systems: rhizotron (black color), container (grey color) and transplanted (hacked) on IAA (A), IBA (B), IA-Ala (C), IA-Leu (D), IA-Phe (E), IA-Me (F) concentration in meristematic zone of medium and long lateral roots of Q. robur seedlings. Fig. S7. The effect of cultivation systems: rhizotron (black color), container (grey color) and transplanted (hacked) on tZ (A), 2iP (B) concentration in meristematic zone of medium and long lateral root of Q. robur seedlings. Fig. S8. The effect of cultivation systems: rhizotron (black color), container (grey color) and transplanted (hacked) on ACC (A), ABA (B), SA (C) concentration in meristematic zone of medium and long lateral roots of Q. robur seedlings. Fig. S9. The effect of cu [file 12870_2024_4797_MOESM1_ESM.docx]

Fig. S1. The effect of cultivation systems: rhizotron (black color), container (grey color) and transplanted (hacked) on IAA (A), IBA (B), IA-Ala (C), IA-Leu (D), IA-Phe (E), IA-Me (F) concentration in elongation zone of short, medium and long taproots of *Q. robur* seedlings. Each point represents the mean hormone values for each root length classes in each cultivation system. Each point represents the mean incorporating multiple individual roots from each cultivation systems. Hormones concentration values were log_10_-transformed before statistical analysis, but figures present non-transformed data. Significance of variation between cultivation systems within length classes i.e. short, medium and long results from an analysis of variance (ANOVA) are given for each length classes panel. Different lower-case letters indicate significantly different means among different cultivation systems within a given length classes at α = 0.05 according to Tukey’s test. Error bars represent the standard error.





Fig. S2. The effect of cultivation systems: rhizotron (black color), container (grey color) and transplanted (hacked) on tZ (A), 2iP (B) concentration in elongation zone of short, medium and long taproots of *Q. robur* seedlings. Each point represents the mean hormone values for each root length classes in each cultivation system. Each point represents the mean incorporating multiple individual roots from each cultivation systems. Hormones concentration values were log_10_-transformed before statistical analysis, but figures present non-transformed data. Significance of variation between cultivation systems within length classes i.e. short, medium and long results from an analysis of variance (ANOVA) are given for each length classes panel. Different lower-case letters indicate significantly different means among different cultivation systems within a given length classes at α = 0.05 according to Tukey’s test. Error bars represent the standard error.





Fig. S3. The effect of cultivation systems: rhizotron (black color), container (grey color) and transplanted (hacked) on ACC (A), ABA (B), SA (C) concentration in elongation zone of short, medium and long taproots of *Q. robur* seedlings. Each point represents the mean hormone values for each root length classes in each cultivation system. Each point represents the mean incorporating multiple individual roots from each cultivation systems. Hormones concentration values were log_10_-transformed before statistical analysis, but figures present non-transformed data. Significance of variation between cultivation systems within length classes i.e. short, medium and long results from an analysis of variance (ANOVA) are given for each length classes panel. Different lower-case letters indicate significantly different means among different cultivation systems within a given length classes at α = 0.05 according to Tukey’s test. Error bars represent the standard error.





Fig. S4. The effect of cultivation systems: rhizotron (black color), container (grey color) and transplanted (hacked) on GA1 (A), GA3 (B), GA4 (C) GA7 (D) concentration in elongation zone of short, medium and long taproots of *Q. robur* seedlings. Each point represents the mean hormone values for each root length classes in each cultivation system. Each point represents the mean incorporating multiple individual roots from each cultivation systems. Hormones concentration values were log_10_-transformed before statistical analysis, but figures present non-transformed data. Significance of variation between cultivation systems within length classes i.e. short, medium and long results from an analysis of variance (ANOVA) are given for each length classes panel. Different lower-case letters indicate significantly different means among different cultivation systems within a given length classes at α = 0.05 according to Tukey’s test. Error bars represent the standard error.





Fig. S5. The effect of cultivation systems: rhizotron (black color), container (grey color) and transplanted (hacked) on JA (A), MeJA (B) concentration in elongation zone of short, medium and long taproots of *Q. robur* seedlings. Each point represents the mean hormone values for each root length classes in each cultivation system. Each point represents the mean incorporating multiple individual roots from each cultivation systems. Hormones concentration values were log_10_-transformed before statistical analysis, but figures present non-transformed data. Significance of variation between cultivation systems within length classes i.e. short, medium and long results from an analysis of variance (ANOVA) are given for each length classes panel. Different lower-case letters indicate significantly different means among different cultivation systems within a given length classes at α = 0.05 according to Tukey’s test. Error bars represent the standard error.





Fig. S6. The effect of cultivation systems: rhizotron (black color), container (grey color) and transplanted (hacked) on IAA (A), IBA (B), IA-Ala (C), IA-Leu (D), IA-Phe (E), IA-Me (F) concentration in meristematic zone of medium and long lateral roots of *Q. robur* seedlings. Each point represents the mean hormone values for each root length classes in each cultivation system. Each point represents the mean incorporating multiple individual roots from each cultivation systems. Hormones concentration values were log_10_-transformed before statistical analysis, but figures present non-transformed data. Significance of variation between cultivation systems within length classes i.e. short, medium and long results from an analysis of variance (ANOVA) are given for each length classes panel. Different lower-case letters indicate significantly different means among different cultivation systems within a given length classes at α = 0.05 according to Tukey’s test. Error bars represent the standard error.





Fig. S7. The effect of cultivation systems: rhizotron (black color), container (grey color) and transplanted (hacked) on tZ (A), 2iP (B) concentration in meristematic zone of medium and long lateral root of *Q. robur* seedlings. Each point represents the mean hormone values for each root length classes in each cultivation system. Each point represents the mean incorporating multiple individual roots from each cultivation systems. Hormones concentration values were log_10_-transformed before statistical analysis, but figures present non-transformed data. Significance of variation between cultivation systems within length classes i.e. short, medium and long results from an analysis of variance (ANOVA) are given for each length classes panel. Different lower-case letters indicate significantly different means among different cultivation systems within a given length classes at α = 0.05 according to Tukey’s test. Error bars represent the standard error.





Fig. S8. The effect of cultivation systems: rhizotron (black color), container (grey color) and transplanted (hacked) on ACC (A), ABA (B), SA (C) concentration in meristematic zone of medium and long lateral roots of *Q. robur* seedlings. Each point represents the mean hormone values for each root length classes in each cultivation system. Each point represents the mean incorporating multiple individual roots from each cultivation systems. Hormones concentration values were log_10_-transformed before statistical analysis, but figures present non-transformed data. Significance of variation between cultivation systems within length classes i.e. short, medium and long results from an analysis of variance (ANOVA) are given for each length classes panel. Different lower-case letters indicate significantly different means among different cultivation systems within a given length classes at α = 0.05 according to Tukey’s test. Error bars represent the standard error.





Fig. S9. The effect of cultivation systems: rhizotron (black color), container (grey color) and transplanted (hacked) on GA1 (A), GA3 (B), GA4 (C) GA7 (D) concentration in meristematic zone of medium and long lateral roots of *Q. robur* seedlings. Each point represents the mean hormone values for each root length classes in each cultivation system. Each point represents the mean incorporating multiple individual roots from each cultivation systems. Hormones concentration values were log_10_-transformed before statistical analysis, but figures present non-transformed data. Significance of variation between cultivation systems within length classes i.e. short, medium and long results from an analysis of variance (ANOVA) are given for each length classes panel. Different lower-case letters indicate significantly different means among different cultivation systems within a given length classes at α = 0.05 according to Tukey’s test. Error bars represent the standard error.





Fig. S10. The effect of cultivation systems: rhizotron (black color), container (grey color) and transplanted (hacked) on JA (A), MeJA (B) concentration in meristematic zone of medium and long lateral roots of *Q. robur* seedlings. Each point represents the mean hormone values for each root length classes in each cultivation system. Each point represents the mean incorporating multiple individual roots from each cultivation systems. Hormones concentration values were log_10_-transformed before statistical analysis, but figures present non-transformed data. Significance of variation between cultivation systems within length classes i.e. short, medium and long results from an analysis of variance (ANOVA) are given for each length classes panel. Different lower-case letters indicate significantly different means among different cultivation systems within a given length classes at α = 0.05 according to Tukey’s test. Error bars represent the standard error.
