# Supplementary material for: Identification of genetics and hormonal factors involved in Quercus robur root growth regulation in different cultivation system
Source: BMC Plant Biol. 2024 Feb 20;24:123. doi: 10.1186/s12870-024-04797-z (PMC10877882; doi:10.1186/s12870-024-04797-z)
Supplement: Supplementary file 2 — Additional file 2: Table S1. MRM values and positive (+) and negative (-) ionization used in the analysis of phytohormones and deuterated standards. [file 12870_2024_4797_MOESM2_ESM.docx]

| Table S1. MRM values and positive (+) and negative (-) ionization used in the analysis of phytohormones and deuterated standards. | | | | |
| --- | --- | --- | --- | --- |
|  |  |  |  |  |
| Hormone | Full name | MRM values and ionization | |  |
|  |  | endogenous phytohormone | deuterated standard |  |
| IAA | indole-3-acetic acid | IAA(+) 176→130 | d2-IAA(+) 178→132 |  |
| IA-Ala | Indole-3-acetyl-L-alanine | IA-Ala(+) 247→130 | d5,15N-IA-Ala(+) 252→134 |  |
| IA-Leu | indole-3-acetyl-L-leucine | IA-Leu(+) 294→134 | d2-IA-Leu(+) 294→134 |  |
| IA-Phe | indole-3-acetyl-L-phenylalanine | IA-Phe(+) 362→130 | d2-IA-Phe(+) 328→134 |  |
| IA-Me | indole-3-acetyl-L-methionine | IAMe(+) 190→130 | d5-IAMe(+) 194→134 |  |
| IBA | indole-3-butyric acid | IBA(+) 204→130 | d2-IBA(+) 206→131 |  |
| tZ | trans-Zeatin | tZ(+) 220→202 | d5-tZ(+) 225→137 |  |
| 2iP | N6-(2-Isopentenyl)adenine | 2iP(+) 204→148 | d5-2iP(+) 210→137 |  |
| ACC | 1-aminocyclopropane-1-carboxylic acid | ACC(+) 102→58 | d2-ACC(+) 106→60 |  |
| ABA | abscisic acid | ABA(-) 263→153 | d5-ABA(-) 269→159 |  |
| SA | salicylic acid | SA(-) 137→93 | d4-SA (-) 141→97 |  |
| GA3 | gibberellins 3 | GA3(-) 345→239 | d2-GA3(-) 347→241 |  |
| GA1 | gibberellins 1 | GA1(-) 347→259 | d2-GA1(-) 349→261 |  |
| GA7 | gibberellins 7 | GA7(-) 329→223 | d2GA7(-) 331→225 |  |
| GA4 | gibberellins 4 | GA4(-) 331→287 | d2-GA4(-) 333→259 |  |
| JA | jasmonic acid | JA(+) 211→133 | d5-JA(+) 214→134 |  |
| MeJA | methyl jasmonate | MeJA(+) 225→151 | d2-JAMe(+) 227→153 |  |
