# Supplementary material for: Identification of genetics and hormonal factors involved in Quercus robur root growth regulation in different cultivation system
Source: BMC Plant Biol. 2024 Feb 20;24:123. doi: 10.1186/s12870-024-04797-z (PMC10877882; doi:10.1186/s12870-024-04797-z)
Supplement: Supplementary file 3 — Additional file 3: Table S2. Differential expression patterns of plant hormone biosynthesis related genes in library comparison between roots in different cultivation systems. [file 12870_2024_4797_MOESM3_ESM.docx]

Table S2. Differential expression patterns of plant hormone biosynthesis related genes in library comparison between roots in different cultivation systems.

| **Comparison** | **Hormone** | **Trasncript ID** | **Gene ID** | **log2FoldChange** | **Symbol** | **Gene description** | **RPKM Transplanted** | **RPKM Container** | **Function** |
| --- | --- | --- | --- | --- | --- | --- | --- | --- | --- |
| STR_C vs. STR_CRH | IAA | MSTRG.11988.1 | MSTRG.11988 | -2,2732 | TAA1 | L-tryptophan--pyruvate aminotransferase 1 | 77,67 | 13,67 | auxin biosynthesis |
| MTR_C vs. MTR_CRH | IAA | MSTRG.9561.1 | MSTRG.9561 | -2,9936 | TAA1 | L-tryptophan--pyruvate aminotransferase 1 | 60,33 | 7,67 | auxin biosynthesis |
| LTR_C vs. LTR_CRH | IAA | MSTRG.11988.1 | MSTRG.11988 | -1,899 | TAA1 | L-tryptophan--pyruvate aminotransferase 1 | 126,33 | 38,67 | auxin biosynthesis |
| MEZ_C vs. MEZ_CRH | IAA | MSTRG.11988.1 | MSTRG.11988 | -3,4452 | TAA1 | L-tryptophan--pyruvate aminotransferase 1 | 7,00 | 0,67 | auxin biosynthesis |
| MLR_C vs. MLR_CRH | IAA | MSTRG.30679.1 | MSTRG.30679 | -1,8779 | YUC6 | Indole-3-pyruvate monooxygenase YUCCA6 | 542,33 | 162,00 | auxin biosynthesis |

| **Comparison** | **Hormone** | **Trasncript ID** | **Gene ID** | **log2FoldChange** | **Symbol** | **Gene description** | **RPKM Transplanted** | **RPKM Rhizotron** | **Function** |
| --- | --- | --- | --- | --- | --- | --- | --- | --- | --- |
| MTR_RH vs. MTR_CRH | IAA | MSTRG.9561.1 | MSTRG.9561 | -2,6868 | TAA1 | L-tryptophan--pyruvate aminotransferase 1 | 60,33 | 9,33 | auxin biosynthesis |
| LTR_RH vs. LTR_CRH | IAA | MSTRG.9561.1 | MSTRG.9561 | -2,8859 | TAA1 | L-tryptophan--pyruvate aminotransferase 1 | 45,33 | 6,00 | auxin biosynthesis |
| MEZ_RH vs. MEZ_CRH | IAA | MSTRG.34007.1 | MSTRG.34007 | -1,9869 | YUC8 | Probable indole-3-pyruvate monooxygenase YUCCA8 | 20,00 | 5,00 | auxin biosynthesis |
| MEZ_RH vs. MEZ_CRH | IAA | MSTRG.11988.1 | MSTRG.11988 | -3,4025 | TAA1 | L-tryptophan--pyruvate aminotransferase 1 | 7,00 | 0,67 | auxin biosynthesis |

| **Comparison** | **Hormone** | **Trasncript ID** | **Gene ID** | **log2FoldChange** | **Symbol** | **Gene description** | RPKM Transplanted | RPKM Container | **Function** |
| --- | --- | --- | --- | --- | --- | --- | --- | --- | --- |
| MLR_C vs. MLR_CRH | CK | MSTRG.19148.1 | MSTRG.19148 | 2,34 | LOG7 | Cytokinin riboside 5'-monophosphate phosphoribohydrolase LOG7 | 7,67 | 43,00 | cytokinin biosynthesis |
| MLR_C vs. MLR_CRH | CK | MSTRG.23760.1 | MSTRG.23760 | 3,0556 | C7351 | Cytokinin hydroxylase | 1,33 | 12,33 | cytokinin biosynthesis |
| LLR_C vs. LLR_CRH | CK | MSTRG.17298.3 | MSTRG.17298 | -1,6816 | ZOG | Zeatin O-glucosyltransferase | 2649,33 | 942,67 | cytokinin biosynthesis |
| LLR_C vs. LLR_CRH | CK | MSTRG.1370.1 | MSTRG.1370 | -1,6328 | LOG1 | Cytokinin riboside 5'-monophosphate phosphoribohydrolase LOG1 | 36,67 | 13,33 | cytokinin biosynthesis |
| STR_C vs. STR_CRH | CK | MSTRG.23763.1 | MSTRG.23763 | 3,3415 | C7351 | Cytokinin hydroxylase | 3,00 | 28,00 | cytokinin biosynthesis |
| LTR_C vs. LTR_CRH | CK | MSTRG.3451.1 | MSTRG.3451 | 1,9203 | LOG3 | Cytokinin riboside 5'-monophosphate phosphoribohydrolase LOG3 | 43,00 | 194,67 | cytokinin biosynthesis |

| **Comparison** | **Hormone** | **Trasncript ID** | **Gene ID** | **log2FoldChange** | **Symbol** | **Gene description** | RPKM Transplanted | RPKM Rhizotron | Function |
| --- | --- | --- | --- | --- | --- | --- | --- | --- | --- |
| MLR_RH vs. MLR_CRH | CK | MSTRG.3451.1 | MSTRG.3451 | 1,5668 | LOG3 | Cytokinin riboside 5'-monophosphate phosphoribohydrolase LOG3 | 90,00 | 267,33 | cytokinin biosynthesis |
| MLR_RH vs. MLR_CRH | CK | MSTRG.19148.1 | MSTRG.19148 | 1,9355 | LOG7 | Cytokinin riboside 5'-monophosphate phosphoribohydrolase LOG7 | 7,67 | 29,67 | cytokinin biosynthesis |
| MLR_RH vs. MLR_CRH | CK | MSTRG.32112.1 | MSTRG.32112 | 1,7977 | IPT | Adenylate isopentenyltransferase | 4,67 | 16,33 | cytokinin biosynthesis |
| LLR_RH vs. LLR_CRH | CK | MSTRG.17298.1 | MSTRG.17298 | -1,9515 | ZOG | Zeatin O-glucosyltransferase | 2649,33 | 798,67 | cytokinin biosynthesis |
| MEZ_RH vs. MEZ_CRH | CK | MSTRG.23763.1 | MSTRG.23763 | 1,7632 | C7351 | Cytokinin hydroxylase | 5,67 | 19,67 | cytokinin biosynthesis |
| STR_RH vs. STR_CRH | CK | MSTRG.23763.1 | MSTRG.23763 | 2,7732 | C7351 | Cytokinin hydroxylase | 3,00 | 19,00 | cytokinin biosynthesis |
| STR_RH vs. STR_CRH | CK | MSTRG.17298.1 | MSTRG.17298 | 2,3155 | ZOG | Zeatin O-glucosyltransferase | 522,33 | 2465,00 | cytokinin biosynthesis |
| MTR_RH vs. MTR_CRH | CK | MSTRG.17298.1 | MSTRG.17298 | 1,7026 | ZOG | Zeatin O-glucosyltransferase | 469,67 | 1527,33 | cytokinin biosynthesis |

| **Comparison** | **Hormone** | **Trasncript ID** | **Gene ID** | **log2FoldChange** | **Symbol** | **Gene description** | **RPKM Container** | **RPKM Rhizotron** | **Function** |
| --- | --- | --- | --- | --- | --- | --- | --- | --- | --- |
| LEZ_RH vs. LEZ_C | CK | MSTRG.5409.1 | MSTRG.5409 | 1,5832 | C7351 | Cytokinin hydroxylase | 41,33 | 113,00 | cytokinin biosynthesis |

| **Comparison** | **Hormone** | **Trasncript ID** | **Gene ID** | **log2FoldChange** | **Symbol** | **Gene description** | **RPKM Transplanted** | **RPKM Container** | **Function** |
| --- | --- | --- | --- | --- | --- | --- | --- | --- | --- |
| MLR_C vs. MLR_CRH | ET | MSTRG.8808.1 | MSTRG.8808 | 1,7462 | ACCH3 | 1-aminocyclopropane-1-carboxylate oxidase homolog | 8,33 | 30,67 | ethylene biosynthesis |
| MLR_C vs. MLR_CRH | ET | MSTRG.17225.1 | MSTRG.17225 | 2,0719 | ACCH1 | 1-aminocyclopropane-1-carboxylate oxidase homolog 1 | 201,67 | 939,00 | ethylene biosynthesis |
| MLR_C vs. MLR_CRH | ET | MSTRG.25190.1 | MSTRG.25190 | 2,0875 | 1A12 | 1-aminocyclopropane-1-carboxylate synthase CMA101 | 8,00 | 37,67 | ethylene biosynthesis |
| MLR_C vs. MLR_CRH | ET | MSTRG.17222.1 | MSTRG.17222 | 2,3439 | ACCH1 | 1-aminocyclopropane-1-carboxylate oxidase homolog 1 | 267,00 | 1494,00 | ethylene biosynthesis |
| MLR_C vs. MLR_CRH | ET | MSTRG.17222.10 | MSTRG.17222 | 2,3439 | ACCH6 | 1-aminocyclopropane-1-carboxylate oxidase homolog 6 | 267,00 | 1494,00 | ethylene biosynthesis |
| MLR_C vs. MLR_CRH | ET | MSTRG.32014.1 | MSTRG.32014 | 2,4175 | 1A1C | 1-aminocyclopropane-1-carboxylate synthase | 36,33 | 214,00 | ethylene biosynthesis |
| MLR_C vs. MLR_CRH | ET | MSTRG.14291.1 | MSTRG.14291 | 2,6614 | 1A1C | 1-aminocyclopropane-1-carboxylate synthase | 18,33 | 128,33 | ethylene biosynthesis |
| MLR_C vs. MLR_CRH | ET | MSTRG.14325.1 | MSTRG.14325 | 3,1908 | 1A1C | 1-aminocyclopropane-1-carboxylate synthase | 1,00 | 10,00 | ethylene biosynthesis |
| MLR_C vs. MLR_CRH | ET | MSTRG.15402.1 | MSTRG.15402 | 4,6245 | ACCH1 | 1-aminocyclopropane-1-carboxylate oxidase homolog 1 | 0,00 | 5,00 | ethylene biosynthesis |
| MLR_C vs. MLR_CRH | ET | MSTRG.17223.1 | MSTRG.17223 | 4,8928 | ACCH1 | 1-aminocyclopropane-1-carboxylate oxidase homolog 1 | 0,00 | 6,00 | ethylene biosynthesis |
| MLR_C vs. MLR_CRH | ET | MSTRG.13653.1 | MSTRG.13653 | 5,8341 | ACCO5 | 1-aminocyclopropane-1-carboxylate oxidase 5 | 49,00 | 3100,33 | ethylene biosynthesis |
| MLR_C vs. MLR_CRH | ET | MSTRG.15403.1 | MSTRG.15403 | 7,589 | ACCH1 | 1-aminocyclopropane-1-carboxylate oxidase homolog 1 | 0,67 | 140,33 | ethylene biosynthesis |
| LLR_C vs. LLR_CRH | ET | MSTRG.5697.1 | MSTRG.5697 | 1,6026 | ACCO1 | 1-aminocyclopropane-1-carboxylate oxidase 1 | 115,67 | 404,33 | ethylene biosynthesis |
| LLR_C vs. LLR_CRH | ET | MSTRG.14325.1 | MSTRG.14325 | 1,7203 | 1A1C | 1-aminocyclopropane-1-carboxylate synthase | 8,00 | 30,67 | ethylene biosynthesis |
| MEZ_C vs. MEZ_CRH | ET | MSTRG.14325.1 | MSTRG.14325 | 1,6454 | 1A1C | 1-aminocyclopropane-1-carboxylate synthase | 16,00 | 52,00 | ethylene biosynthesis |
| MEZ_C vs. MEZ_CRH | ET | MSTRG.24860.1 | MSTRG.24860 | 2,1829 | ACCH1 | 1-aminocyclopropane-1-carboxylate oxidase homolog 1 | 9,33 | 44,00 | ethylene biosynthesis |
| MEZ_C vs. MEZ_CRH | ET | MSTRG.13653.1 | MSTRG.13653 | 3,1066 | ACCO5 | 1-aminocyclopropane-1-carboxylate oxidase 5 | 59,33 | 546,33 | ethylene biosynthesis |
| STR_C vs. STR_CRH | ET | MSTRG.15408.1 | MSTRG.15408 | 1,7855 | ACCH3 | 1-aminocyclopropane-1-carboxylate oxidase homolog | 6,33 | 19,67 | ethylene biosynthesis |
| STR_C vs. STR_CRH | ET | MSTRG.11166.1 | MSTRG.11166 | 1,9514 | 1A11 | 1-aminocyclopropane-1-carboxylate synthase 1 | 27,00 | 94,00 | ethylene biosynthesis |
| STR_C vs. STR_CRH | ET | MSTRG.14325.1 | MSTRG.14325 | 2,4896 | 1A1C | 1-aminocyclopropane-1-carboxylate synthase | 15,00 | 77,67 | ethylene biosynthesis |
| STR_C vs. STR_CRH | ET | MSTRG.15402.1 | MSTRG.15402 | 3,4001 | ACCH1 | 1-aminocyclopropane-1-carboxylate oxidase homolog 1 | 5,67 | 53,33 | ethylene biosynthesis |
| MTR_C vs. MTR_CRH | ET | MSTRG.13876.1 | MSTRG.13876 | 1,5208 | ACCO | 1-aminocyclopropane-1-carboxylate oxidase | 188,00 | 559,00 | ethylene biosynthesis |
| MTR_C vs. MTR_CRH | ET | MSTRG.15404.1 | MSTRG.15404 | 2,3528 | ACCH1 | 1-aminocyclopropane-1-carboxylate oxidase homolog 1 | 16,00 | 85,33 | ethylene biosynthesis |
| LTR_C vs. LTR_CRH | ET | MSTRG.14325.1 | MSTRG.14325 | 1,5945 | 1A1C | 1-aminocyclopropane-1-carboxylate synthase | 22,67 | 82,67 | ethylene biosynthesis |
| LTR_C vs. LTR_CRH | ET | MSTRG.11166.1 | MSTRG.11166 | 1,9149 | 1A11 | 1-aminocyclopropane-1-carboxylate synthase 1 | 34,33 | 154,67 | ethylene biosynthesis |
| LTR_C vs. LTR_CRH | ET | MSTRG.17227.1 | MSTRG.17227 | 2,1644 | ACH11 | 1-aminocyclopropane-1-carboxylate oxidase homolog 11 | 9,33 | 51,67 | ethylene biosynthesis |

| **Comparison** | **Hormone** | **Trasncript ID** | **Gene ID** | **log2FoldChange** | **Symbol** | **Gene description** | **RPKM Transplanted** | **RPKM Rhizotron** | **Function** |
| --- | --- | --- | --- | --- | --- | --- | --- | --- | --- |
| MLR_RH vs. MLR_CRH | ET | MSTRG.5763.1 | MSTRG.5763 | 1,5732 | 1A17 | 1-aminocyclopropane-1-carboxylate synthase 7 | 81,00 | 242,33 | ethylene biosynthesis |
| MLR_RH vs. MLR_CRH | ET | MSTRG.14291.1 | MSTRG.14291 | 1,8214 | 1A1C | 1-aminocyclopropane-1-carboxylate synthase | 18,33 | 65,00 | ethylene biosynthesis |
| MLR_RH vs. MLR_CRH | ET | MSTRG.8809.1 | MSTRG.8809 | 1,9822 | ACCH4 | 1-aminocyclopropane-1-carboxylate oxidase homolog 4 | 105,33 | 417,00 | ethylene biosynthesis |
| MLR_RH vs. MLR_CRH | ET | MSTRG.24860.1 | MSTRG.24860 | 2,0684 | ACCH1 | 1-aminocyclopropane-1-carboxylate oxidase homolog 1 | 61,00 | 257,00 | ethylene biosynthesis |
| MLR_RH vs. MLR_CRH | ET | MSTRG.13876.1 | MSTRG.13876 | 2,1981 | ACCO | 1-aminocyclopropane-1-carboxylate oxidase | 250,67 | 1153,00 | ethylene biosynthesis |
| MLR_RH vs. MLR_CRH | ET | MSTRG.8808.1 | MSTRG.8808 | 2,703 | ACCH3 | 1-aminocyclopropane-1-carboxylate oxidase homolog | 8,33 | 55,00 | ethylene biosynthesis |
| MLR_RH vs. MLR_CRH | ET | MSTRG.8814.1 | MSTRG.8814 | 2,7361 | ACCH3 | 1-aminocyclopropane-1-carboxylate oxidase homolog 3 | 1,00 | 6,67 | ethylene biosynthesis |
| MLR_RH vs. MLR_CRH | ET | MSTRG.14325.1 | MSTRG.14325 | 3,7382 | 1A1C | 1-aminocyclopropane-1-carboxylate synthase | 1,00 | 13,33 | ethylene biosynthesis |
| MLR_RH vs. MLR_CRH | ET | MSTRG.17223.1 | MSTRG.17223 | 4,0255 | ACCH1 | 1-aminocyclopropane-1-carboxylate oxidase homolog 1 | 0,00 | 3,00 | ethylene biosynthesis |
| MLR_RH vs. MLR_CRH | ET | MSTRG.13653.1 | MSTRG.13653 | 5,8244 | ACCO5 | 1-aminocyclopropane-1-carboxylate oxidase 5 | 49,00 | 2789,00 | ethylene biosynthesis |
| MLR_RH vs. MLR_CRH | ET | MSTRG.15403.1 | MSTRG.15403 | 7,968 | ACCH1 | 1-aminocyclopropane-1-carboxylate oxidase homolog 1 | 0,67 | 167,00 | ethylene biosynthesis |
| MEZ_RH vs. MEZ_CRH | ET | MSTRG.32014.1 | MSTRG.32014 | 1,5757 | 1A1C | 1-aminocyclopropane-1-carboxylate synthase | 181,00 | 549,33 | ethylene biosynthesis |
| MEZ_RH vs. MEZ_CRH | ET | MSTRG.24860.1 | MSTRG.24860 | 2,7359 | ACCH1 | 1-aminocyclopropane-1-carboxylate oxidase homolog 1 | 9,33 | 62,67 | ethylene biosynthesis |
| MEZ_RH vs. MEZ_CRH | ET | MSTRG.13653.1 | MSTRG.13653 | 3,3332 | ACCO5 | 1-aminocyclopropane-1-carboxylate oxidase 5 | 59,33 | 607,33 | ethylene biosynthesis |
| STR_RH vs. STR_CRH | ET | MSTRG.11166.1 | MSTRG.11166 | 1,8609 | 1A11 | 1-aminocyclopropane-1-carboxylate synthase 1 | 27,00 | 94,00 | ethylene biosynthesis |
| STR_RH vs. STR_CRH | ET | MSTRG.5697.1 | MSTRG.5697 | 1,9471 | ACCO1 | 1-aminocyclopropane-1-carboxylate oxidase 1 | 495,00 | 1820,67 | ethylene biosynthesis |
| STR_RH vs. STR_CRH | ET | MSTRG.14325.1 | MSTRG.14325 | 2,1543 | 1A1C | 1-aminocyclopropane-1-carboxylate synthase | 15,00 | 63,67 | ethylene biosynthesis |
| STR_RH vs. STR_CRH | ET | MSTRG.15402.1 | MSTRG.15402 | 2,1695 | ACCH1 | 1-aminocyclopropane-1-carboxylate oxidase homolog 1 | 5,67 | 24,00 | ethylene biosynthesis |
| MTR_RH vs. MTR_CRH | ET | MSTRG.15404.1 | MSTRG.15404 | 2,7644 | ACCH1 | 1-aminocyclopropane-1-carboxylate oxidase homolog 1 | 16,00 | 109,00 | ethylene biosynthesis |
| LTR_RH vs. LTR_CRH | ET | MSTRG.15400.1 | MSTRG.15400 | 1,7854 | ACCH1 | 1-aminocyclopropane-1-carboxylate oxidase homolog 1 | 65,00 | 227,33 | ethylene biosynthesis |
| **Comparison** | **Hormone** | **Trasncript ID** | **Gene ID** | **log2FoldChange** | **Symbol** | **Gene description** | **RPKM Container** | **RPKM Rhizotron** | **Function** |
| MLR_RH vs. MLR_C | ET | MSTRG.15402.1 | MSTRG.15402 | -4,6209 | ACCH1 | 1-aminocyclopropane-1-carboxylate oxidase homolog 1 | 5,00 | 0,00 | ethylene biosynthesis |
| MLR_RH vs. MLR_C | ET | MSTRG.5769.1 | MSTRG.5769 | -3,0655 | 1A17 | 1-aminocyclopropane-1-carboxylate synthase 7 | 9,33 | 1,00 | ethylene biosynthesis |
| MLR_RH vs. MLR_C | ET | MSTRG.17225.1 | MSTRG.17225 | -2,7369 | ACCH1 | 1-aminocyclopropane-1-carboxylate oxidase homolog 1 | 939,00 | 127,00 | ethylene biosynthesis |
| MLR_RH vs. MLR_C | ET | MSTRG.17227.1 | MSTRG.17227 | -1,8917 | ACH11 | 1-aminocyclopropane-1-carboxylate oxidase homolog 11 | 16,33 | 4,00 | ethylene biosynthesis |
| MLR_RH vs. MLR_C | ET | MSTRG.32014.1 | MSTRG.32014 | -1,6316 | 1A1C | 1-aminocyclopropane-1-carboxylate synthase | 214,00 | 62,00 | ethylene biosynthesis |
| MLR_RH vs. MLR_C | ET | MSTRG.11166.1 | MSTRG.11166 | -1,5633 | 1A11 | 1-aminocyclopropane-1-carboxylate synthase 1 | 63,00 | 19,67 | ethylene biosynthesis |
| LLR_RH vs. LLR_C | ET | MSTRG.17227.1 | MSTRG.17227 | -4,2357 | ACH11 | 1-aminocyclopropane-1-carboxylate oxidase homolog 11 | 6,67 | 0,33 | ethylene biosynthesis |
| LEZ_RH vs. LEZ_C | ET | MSTRG.6777.1 | MSTRG.6777 | 1,595 | ACCO | 1-aminocyclopropane-1-carboxylate oxidase | 20,67 | 57,67 | ethylene biosynthesis |
| LEZ_RH vs. LEZ_C | ET | MSTRG.14291.1 | MSTRG.14291 | 2,1246 | 1A1C | 1-aminocyclopropane-1-carboxylate synthase | 157,00 | 596,67 | ethylene biosynthesis |
| LEZ_RH vs. LEZ_C | ET | MSTRG.32014.1 | MSTRG.32014 | 2,3005 | 1A1C | 1-aminocyclopropane-1-carboxylate synthase | 202,33 | 875,67 | ethylene biosynthesis |
| STR_RH vs. STR_C | ET | MSTRG.25190.1 | MSTRG.25190 | 1,5638 | 1A12 | 1-aminocyclopropane-1-carboxylate synthase CMA101 | 29,00 | 95,00 | ethylene biosynthesis |
| MTR_RH vs. MTR_C | ET | MSTRG.14291.1 | MSTRG.14291 | -2,0497 | 1A1C | 1-aminocyclopropane-1-carboxylate synthase | 360,67 | 83,33 | ethylene biosynthesis |
| MTR_RH vs. MTR_C | ET | MSTRG.11166.1 | MSTRG.11166 | -1,8753 | 1A11 | 1-aminocyclopropane-1-carboxylate synthase 1 | 56,67 | 15,33 | ethylene biosynthesis |
| MTR_RH vs. MTR_C | ET | MSTRG.32014.1 | MSTRG.32014 | -1,8003 | 1A1C | 1-aminocyclopropane-1-carboxylate synthase | 362,67 | 99,33 | ethylene biosynthesis |

| **Comparison** | **Hormone** | **Trasncript ID** | **Gene ID** | **log2FoldChange** | **Symbol** | **Gene description** | **RPKM Transplanted** | **RPKM Rhizotron** | **Function** |
| --- | --- | --- | --- | --- | --- | --- | --- | --- | --- |
| MEZ_RH vs. MEZ_CRH | ABA | MSTRG.13453.1 | MSTRG.13453 | -2,4408 | NCED1 | 9-cis-epoxycarotenoid dioxygenase NCED1 | 2309,00 | 432,33 | abscisc acid biosynthesis |
| MEZ_RH vs. MEZ_CRH | ABA | MSTRG.32782.1 | MSTRG.32782 | -2,2765 | NCED1 | 9-cis-epoxycarotenoid dioxygenase NCED1 | 120,67 | 25,33 | abscisc acid biosynthesis |
| LEZ_RH vs. LEZ_CRH | ABA | MSTRG.32782.1 | MSTRG.32782 | -2,5658 | NCED1 | 9-cis-epoxycarotenoid dioxygenase NCED1 | 348,33 | 62,33 | abscisc acid biosynthesis |

| **Comparison** | **Hormone** | **Trasncript ID** | **Gene ID** | **log2FoldChange** | **Symbol** | **Gene description** | **RPKM Container** | **RPKM Rhizotron** | **Function** |
| --- | --- | --- | --- | --- | --- | --- | --- | --- | --- |
| LLR_RH vs. LLR_C | ABA | MSTRG.32782.1 | MSTRG.32782 | -2,2745 | NCED1 | 9-cis-epoxycarotenoid dioxygenase NCED1 | 405,67 | 84,67 | abscisc acid biosynthesis |
| LLR_RH vs. LLR_C | ABA | MSTRG.13453.1 | MSTRG.13453 | -2,0892 | NCED1 | 9-cis-epoxycarotenoid dioxygenase NCED1 | 2557,00 | 609,33 | abscisc acid biosynthesis |
| LEZ_RH vs. LEZ_C | ABA | MSTRG.13453.1 | MSTRG.13453 | -2,1878 | NCED1 | 9-cis-epoxycarotenoid dioxygenase NCED1 | 4296,00 | 890,67 | abscisc acid biosynthesis |
| LTR_RH vs. LTR_C | ABA | MSTRG.13453.1 | MSTRG.13453 | -2,4219 | NCED1 | 9-cis-epoxycarotenoid dioxygenase NCED1 | 1796,67 | 278,67 | abscisc acid biosynthesis |

| **Comparison** | **Hormone** | **Trasncript ID** | **Gene ID** | **log2FoldChange** | **Symbol** | **Gene description** | **RPKM Transplanted** | **RPKM Container** | **Function** |
| --- | --- | --- | --- | --- | --- | --- | --- | --- | --- |
| MLR_C vs. MLR_CRH | GA | MSTRG.33954.1 | MSTRG.33954 | 2,2458 | G2OX2 | Gibberellin 2-beta-dioxygenase 2 | 42,33 | 219,33 | gibberellin biosynthesis |
| MLR_C vs. MLR_CRH | GA | MSTRG.23935.1 | MSTRG.23935 | 2,3816 | GAOX2 | Gibberellin 20 oxidase 2 | 24,33 | 140,67 | gibberellin biosynthesis |
| MLR_C vs. MLR_CRH | GA | MSTRG.13536.1 | MSTRG.13536 | 5,0469 | GAOX2 | Gibberellin 20 oxidase 2 | 0,67 | 24,33 | gibberellin biosynthesis |
| LLR_C vs. LLR_CRH | GA | MSTRG.22681.1 | MSTRG.22681 | 2,0065 | G3OX | Gibberellin 3-beta-dioxygenase 1 | 6,67 | 31,00 | gibberellin biosynthesis |
| LEZ_C vs. LEZ_CRH | GA | MSTRG.31144.1 | MSTRG.31144 | -1,9939 | G3OX | Gibberellin 3-beta-dioxygenase 1 | 142,00 | 39,00 | gibberellin biosynthesis |
| LEZ_C vs. LEZ_CRH | GA | MSTRG.9123.1 | MSTRG.9123 | -1,696 | G2OX6 | Gibberellin 2-beta-dioxygenase 6 | 38,33 | 13,33 | gibberellin biosynthesis |
| STR_C vs. STR_CRH | GA | MSTRG.22681.1 | MSTRG.22681 | 3,9855 | G3OX | Gibberellin 3-beta-dioxygenase 1 | 23,33 | 306,33 | gibberellin biosynthesis |
| MTR_C vs. MTR_CRH | GA | MSTRG.22681.1 | MSTRG.22681 | 2,4425 | G3OX | Gibberellin 3-beta-dioxygenase 1 | 39,33 | 220,00 | gibberellin biosynthesis |
| MTR_C vs. MTR_CRH | GA | MSTRG.32735.1 | MSTRG.32735 | -1,9282 | KO1 | Ent-kaurene oxidase | 219,00 | 60,00 | gibberellin biosynthesis |
| LTR_C vs. LTR_CRH | GA | MSTRG.33954.1 | MSTRG.33954 | 1,9776 | G2OX2 | Gibberellin 2-beta-dioxygenase 2 | 74,33 | 341,67 | gibberellin biosynthesis |

| **Comparison** | **Hormone** | **Trasncript ID** | **Gene ID** | **log2FoldChange** | **Symbol** | **Gene description** | **RPKM Transplanted** | **RPKM Rhizotron** | **Function** |
| --- | --- | --- | --- | --- | --- | --- | --- | --- | --- |
| MLR_RH vs. MLR_CRH | GA | MSTRG.31144.1 | MSTRG.31144 | 1,6723 | G3OX | Gibberellin 3-beta-dioxygenase 1 | 134,33 | 430,33 | gibberellin biosynthesis |
| MLR_RH vs. MLR_CRH | GA | MSTRG.13439.1 | MSTRG.13439 | 1,9892 | G2OX1 | Gibberellin 2-beta-dioxygenase 1 | 271,33 | 1080,00 | gibberellin biosynthesis |
| MLR_RH vs. MLR_CRH | GA | MSTRG.33954.1 | MSTRG.33954 | 2,4759 | G2OX2 | Gibberellin 2-beta-dioxygenase 2 | 42,33 | 237,00 | gibberellin biosynthesis |
| LLR_RH vs. LLR_CRH | GA | MSTRG.32736.1 | MSTRG.32736 | 1,8664 | KO1 | Ent-kaurene oxidase | 27,67 | 118,00 | gibberellin biosynthesis |
| LLR_RH vs. LLR_CRH | GA | MSTRG.22681.1 | MSTRG.22681 | 1,995 | G3OX | Gibberellin 3-beta-dioxygenase 1 | 6,67 | 31,33 | gibberellin biosynthesis |
| MEZ_RH vs. MEZ_CRH | GA | MSTRG.15029.1 | MSTRG.15029 | -2,6083 | G2OX2 | Gibberellin 2-beta-dioxygenase 2 | 74,00 | 12,33 | gibberellin biosynthesis |
| MEZ_RH vs. MEZ_CRH | GA | MSTRG.9123.1 | MSTRG.9123 | -1,6949 | G2OX6 | Gibberellin 2-beta-dioxygenase 6 | 52,33 | 16,33 | gibberellin biosynthesis |
| STR_RH vs. STR_CRH | GA | MSTRG.20624.1 | MSTRG.20624 | 1,6516 | GAOXL | Gibberellin 20-oxidase-like protein | 63,33 | 187,33 | gibberellin biosynthesis |
| STR_RH vs. STR_CRH | GA | MSTRG.22681.1 | MSTRG.22681 | 3,3931 | G3OX | Gibberellin 3-beta-dioxygenase 1 | 23,33 | 233,33 | gibberellin biosynthesis |
| MTR_RH vs. MTR_CRH | GA | MSTRG.22681.1 | MSTRG.22681 | 2,6425 | G3OX | Gibberellin 3-beta-dioxygenase 1 | 39,33 | 243,67 | gibberellin biosynthesis |
| LTR_RH vs. LTR_CRH | GA | MSTRG.22681.1 | MSTRG.22681 | 2,1804 | G3OX | Gibberellin 3-beta-dioxygenase 1 | 25,67 | 120,67 | gibberellin biosynthesis |

| **Comparison** | **Hormone** | **Trasncript ID** | **Gene ID** | **log2FoldChange** | **Symbol** | **Gene description** | **RPKM Container** | **RPKM Rhizotron** | **Function** |
| --- | --- | --- | --- | --- | --- | --- | --- | --- | --- |
| MLR_RH vs. MLR_C | GA | MSTRG.13536.1 | MSTRG.13536 | -5,0455 | GAOX2 | Gibberellin 20 oxidase 2 | 24,33 | 0,67 | gibberellin biosynthesis |
| LTR_RH vs. LTR_C | GA | MSTRG.15029.1 | MSTRG.15029 | -2,4024 | G2OX2 | Gibberellin 2-beta-dioxygenase 2 | 23,00 | 3,67 | gibberellin biosynthesis |
| LTR_RH vs. LTR_C | GA | MSTRG.33954.1 | MSTRG.33954 | -2,1981 | G2OX2 | Gibberellin 2-beta-dioxygenase 2 | 341,67 | 62,33 | gibberellin biosynthesis |
| LTR_RH vs. LTR_C | GA | MSTRG.9123.1 | MSTRG.9123 | -2,0415 | G2OX6 | Gibberellin 2-beta-dioxygenase 6 | 81,00 | 16,67 | gibberellin biosynthesis |

| **Comparison** | **Hormone** | **Trasncript ID** | **Gene ID** | **log2FoldChange** | **Symbol** | **Gene description** | **RPKM Transplanted** | **RPKM Container** | **Function** |
| --- | --- | --- | --- | --- | --- | --- | --- | --- | --- |
| MLR_C vs. MLR_CRH | JA | MSTRG.424.1 | MSTRG.424 | 5,0547 | LOX21 | Linoleate 13S-lipoxygenase 2-1 | 1,33 | 49,67 | jasmonate biosynthesis |
| MLR_C vs. MLR_CRH | JA | MSTRG.34848.1 | MSTRG.34848 | 6,176 | LOX21 | Linoleate 13S-lipoxygenase 2-1 | 0,00 | 14,33 | jasmonate biosynthesis |
| MLR_C vs. MLR_CRH | JA | MSTRG.572.1 | MSTRG.572 | 6,3809 | LOX21 | Linoleate 13S-lipoxygenase 2-1 | 0,00 | 16,67 | jasmonate biosynthesis |
| MLR_C vs. MLR_CRH | JA | MSTRG.575.1 | MSTRG.575 | 7,1496 | LOX21 | Linoleate 13S-lipoxygenase 2-1 | 0,00 | 28,33 | jasmonate biosynthesis |
| MLR_C vs. MLR_CRH | JA | MSTRG.34849.1 | MSTRG.34849 | 8,2276 | LOX21 | Linoleate 13S-lipoxygenase 2-1 | 0,00 | 59,67 | jasmonate biosynthesis |
| MLR_C vs. MLR_CRH | JA | MSTRG.3282.1 | MSTRG.3282 | 5,9236 | OPR1 | 12-oxophytodienoate reductase 1 | 0,00 | 12,33 | jasmonate biosynthesis |
| LLR_C vs. LLR_CRH | JA | MSTRG.424.1 | MSTRG.424 | 1,5328 | LOX21 | Linoleate 13S-lipoxygenase 2-1 | 25,33 | 84,33 | jasmonate biosynthesis |
| LLR_C vs. LLR_CRH | JA | MSTRG.34849.1 | MSTRG.34849 | 1,9076 | LOX21 | Linoleate 13S-lipoxygenase 2-1 | 21,67 | 90,33 | jasmonate biosynthesis |
| LLR_C vs. LLR_CRH | JA | MSTRG.570.1 | MSTRG.570 | 2,0014 | LOX21 | Linoleate 13S-lipoxygenase 2-1 | 20,33 | 93,00 | jasmonate biosynthesis |
| LLR_C vs. LLR_CRH | JA | MSTRG.28842.1 | MSTRG.28842 | 2,1471 | AOS3 | Allene oxide synthase 3 | 4,67 | 23,33 | jasmonate biosynthesis |
| MEZ_C vs. MEZ_CRH | JA | MSTRG.424.1 | MSTRG.424 | 6,2907 | LOX21 | Linoleate 13S-lipoxygenase 2-1 | 5,00 | 400,67 | jasmonate biosynthesis |
| LEZ_C vs. LEZ_CRH | JA | MSTRG.34851.1 | MSTRG.34851 | 1,8815 | LOX21 | Linoleate 13S-lipoxygenase 2-1 | 80,00 | 336,33 | jasmonate biosynthesis |
| LEZ_C vs. LEZ_CRH | JA | MSTRG.424.1 | MSTRG.424 | 2,1204 | LOX21 | Linoleate 13S-lipoxygenase 2-1 | 151,67 | 733,00 | jasmonate biosynthesis |
| LEZ_C vs. LEZ_CRH | JA | MSTRG.34849.1 | MSTRG.34849 | 4,0821 | LOX21 | Linoleate 13S-lipoxygenase 2-1 | 3,33 | 64,33 | jasmonate biosynthesis |
| LEZ_C vs. LEZ_CRH | JA | MSTRG.570.1 | MSTRG.570 | 4,3374 | LOX21 | Linoleate 13S-lipoxygenase 2-1 | 2,00 | 45,33 | jasmonate biosynthesis |
| LEZ_C vs. LEZ_CRH | JA | MSTRG.34848.1 | MSTRG.34848 | 4,5201 | LOX21 | Linoleate 13S-lipoxygenase 2-1 | 0,67 | 17,67 | jasmonate biosynthesis |
| LEZ_C vs. LEZ_CRH | JA | MSTRG.572.1 | MSTRG.572 | 4,6844 | LOX21 | Linoleate 13S-lipoxygenase 2-1 | 0,67 | 19,00 | jasmonate biosynthesis |
| LEZ_C vs. LEZ_CRH | JA | MSTRG.575.1 | MSTRG.575 | 5,8275 | LOX21 | Linoleate 13S-lipoxygenase 2-1 | 0,33 | 23,00 | jasmonate biosynthesis |
| STR_C vs. STR_CRH | JA | MSTRG.24102.1 | MSTRG.24102 | 1,9919 | OPR2 | 12-oxophytodienoate reductase 2 | 70,00 | 255,33 | jasmonate biosynthesis |
| MTR_C vs. MTR_CRH | JA | MSTRG.24102.1 | MSTRG.24102 | 1,9166 | OPR2 | 12-oxophytodienoate reductase 2 | 68,00 | 268,00 | jasmonate biosynthesis |
| LTR_C vs. LTR_CRH | JA | MSTRG.570.1 | MSTRG.570 | 5,3367 | LOX21 | Linoleate 13S-lipoxygenase 2-1 | 0,00 | 9,00 | jasmonate biosynthesis |
| LTR_C vs. LTR_CRH | JA | MSTRG.7230.1 | MSTRG.7230 | 1,769 | OPR2 | 12-oxophytodienoate reductase 2 | 85,33 | 362,33 | jasmonate biosynthesis |
| LTR_C vs. LTR_CRH | JA | MSTRG.24084.1 | MSTRG.24084 | 2,3973 | OPR2 | 12-oxophytodienoate reductase 2 | 230,67 | 1456,00 | jasmonate biosynthesis |

| **Comparison** | **Hormone** | **Trasncript ID** | **Gene ID** | **log2FoldChange** | **Symbol** | **Gene description** | **RPKM Transplanted** | **RPKM Rhizotron** | **Function** |
| --- | --- | --- | --- | --- | --- | --- | --- | --- | --- |
| MLR_RH vs. MLR_CRH | JA | MSTRG.28846.1 | MSTRG.28846 | 2,2599 | AOS3 | Allene oxide synthase 3 | 2,00 | 9,67 | jasmonate biosynthesis |
| MLR_RH vs. MLR_CRH | JA | MSTRG.28845.1 | MSTRG.28845 | 2,5742 | AOS3 | Allene oxide synthase 3 | 1150,33 | 6870,33 | jasmonate biosynthesis |
| MLR_RH vs. MLR_CRH | JA | MSTRG.9496.1 | MSTRG.9496 | 2,8 | AOS3 | Allene oxide synthase 3 | 120,33 | 840,33 | jasmonate biosynthesis |
| MLR_RH vs. MLR_CRH | JA | MSTRG.424.1 | MSTRG.424 | 4,5847 | LOX21 | Linoleate 13S-lipoxygenase 2-1 | 1,33 | 32,33 | jasmonate biosynthesis |
| MLR_RH vs. MLR_CRH | JA | MSTRG.570.1 | MSTRG.570 | 6,2556 | LOX21 | Linoleate 13S-lipoxygenase 2-1 | 0,33 | 27,67 | jasmonate biosynthesis |
| MLR_RH vs. MLR_CRH | JA | MSTRG.34848.1 | MSTRG.34848 | 6,2793 | LOX21 | Linoleate 13S-lipoxygenase 2-1 | 0,00 | 14,33 | jasmonate biosynthesis |
| MLR_RH vs. MLR_CRH | JA | MSTRG.572.1 | MSTRG.572 | 6,3596 | LOX21 | Linoleate 13S-lipoxygenase 2-1 | 0,00 | 15,00 | jasmonate biosynthesis |
| MLR_RH vs. MLR_CRH | JA | MSTRG.575.1 | MSTRG.575 | 6,8587 | LOX21 | Linoleate 13S-lipoxygenase 2-1 | 0,00 | 21,33 | jasmonate biosynthesis |
| MLR_RH vs. MLR_CRH | JA | MSTRG.34849.1 | MSTRG.34849 | 8,0917 | LOX21 | Linoleate 13S-lipoxygenase 2-1 | 0,00 | 50,67 | jasmonate biosynthesis |
| LLR_RH vs. LLR_CRH | JA | MSTRG.28842.1 | MSTRG.28842 | 1,9528 | AOS3 | Allene oxide synthase 3 | 4,67 | 21,00 | jasmonate biosynthesis |
| MEZ_RH vs. MEZ_CRH | JA | MSTRG.34849.1 | MSTRG.34849 | 4,3383 | LOX21 | Linoleate 13S-lipoxygenase 2-1 | 1,33 | 27,00 | jasmonate biosynthesis |
| MEZ_RH vs. MEZ_CRH | JA | MSTRG.424.1 | MSTRG.424 | 4,9347 | LOX21 | Linoleate 13S-lipoxygenase 2-1 | 5,00 | 154,67 | jasmonate biosynthesis |
| MEZ_RH vs. MEZ_CRH | JA | MSTRG.575.1 | MSTRG.575 | 5,05 | LOX21 | Linoleate 13S-lipoxygenase 2-1 | 0,00 | 6,33 | jasmonate biosynthesis |
| STR_RH vs. STR_CRH | JA | MSTRG.34851.1 | MSTRG.34851 | 2,5359 | LOX21 | Linoleate 13S-lipoxygenase 2-1 | 20,33 | 113,00 | jasmonate biosynthesis |
| STR_RH vs. STR_CRH | JA | MSTRG.24102.1 | MSTRG.24102 | 2,0691 | OPR2 | 12-oxophytodienoate reductase 2 | 70,00 | 278,33 | jasmonate biosynthesis |
| MTR_RH vs. MTR_CRH | JA | MSTRG.34849.1 | MSTRG.34849 | 3,7277 | LOX21 | Linoleate 13S-lipoxygenase 2-1 | 0,33 | 4,67 | jasmonate biosynthesis |
| MTR_RH vs. MTR_CRH | JA | MSTRG.424.1 | MSTRG.424 | 3,929 | LOX21 | Linoleate 13S-lipoxygenase 2-1 | 36,67 | 559,67 | jasmonate biosynthesis |
| MTR_RH vs. MTR_CRH | JA | MSTRG.24102.1 | MSTRG.24102 | 1,8333 | OPR2 | 12-oxophytodienoate reductase 2 | 68,00 | 241,00 | jasmonate biosynthesis |

| **Comparison** | **Hormone** | **Trasncript ID** | **Gene ID** | **log2FoldChange** | **Symbol** | **Gene description** | RPKM Container | RPKM Rhizotron | Function |
| --- | --- | --- | --- | --- | --- | --- | --- | --- | --- |
| MLR_RH vs. MLR_C | JA | MSTRG.24102.1 | MSTRG.24102 | -2,0239 | OPR2 | 12-oxophytodienoate reductase 2 | 1067,67 | 240,00 | jasmonate biosynthesis |
| LLR_RH vs. LLR_C | JA | MSTRG.424.1 | MSTRG.424 | -1,7244 | LOX21 | Linoleate 13S-lipoxygenase 2-1 | 84,33 | 26,00 | jasmonate biosynthesis |
| LLR_RH vs. LLR_C | JA | MSTRG.7230.1 | MSTRG.7230 | -2,1301 | OPR2 | 12-oxophytodienoate reductase 2 | 39,67 | 9,33 | jasmonate biosynthesis |
| MEZ_RH vs. MEZ_C | JA | MSTRG.34849.1 | MSTRG.34849 | 4,8177 | LOX21 | Linoleate 13S-lipoxygenase 2-1 | 1,00 | 27,00 | jasmonate biosynthesis |
| LEZ_RH vs. LEZ_C | JA | MSTRG.575.1 | MSTRG.575 | -4,979 | LOX21 | Linoleate 13S-lipoxygenase 2-1 | 23,00 | 0,67 | jasmonate biosynthesis |
| LEZ_RH vs. LEZ_C | JA | MSTRG.34849.1 | MSTRG.34849 | -4,6594 | LOX21 | Linoleate 13S-lipoxygenase 2-1 | 64,33 | 2,33 | jasmonate biosynthesis |
| LEZ_RH vs. LEZ_C | JA | MSTRG.34848.1 | MSTRG.34848 | -4,599 | LOX21 | Linoleate 13S-lipoxygenase 2-1 | 17,67 | 0,67 | jasmonate biosynthesis |
| LEZ_RH vs. LEZ_C | JA | MSTRG.570.1 | MSTRG.570 | -4,3795 | LOX21 | Linoleate 13S-lipoxygenase 2-1 | 45,33 | 2,00 | jasmonate biosynthesis |
| LEZ_RH vs. LEZ_C | JA | MSTRG.572.1 | MSTRG.572 | -3,7326 | LOX21 | Linoleate 13S-lipoxygenase 2-1 | 19,00 | 1,33 | jasmonate biosynthesis |
| LEZ_RH vs. LEZ_C | JA | MSTRG.34851.1 | MSTRG.34851 | -2,7888 | LOX21 | Linoleate 13S-lipoxygenase 2-1 | 336,33 | 45,33 | jasmonate biosynthesis |
| LEZ_RH vs. LEZ_C | JA | MSTRG.424.1 | MSTRG.424 | -2,0554 | LOX21 | Linoleate 13S-lipoxygenase 2-1 | 733,00 | 165,00 | jasmonate biosynthesis |
| STR_RH vs. STR_C | JA | MSTRG.9496.1 | MSTRG.9496 | 1,9554 | AOS3 | Allene oxide synthase 3 | 55,00 | 224,67 | jasmonate biosynthesis |
| STR_RH vs. STR_C | JA | MSTRG.28842.1 | MSTRG.28842 | 2,3178 | AOS3 | Allene oxide synthase 3 | 24,67 | 132,67 | jasmonate biosynthesis |
| LTR_RH vs. LTR_C | JA | MSTRG.570.1 | MSTRG.570 | -5,3356 | LOX21 | Linoleate 13S-lipoxygenase 2-1 | 9,00 | 0,00 | jasmonate biosynthesis |
| LTR_RH vs. LTR_C | JA | MSTRG.34849.1 | MSTRG.34849 | -4,6275 | LOX21 | Linoleate 13S-lipoxygenase 2-1 | 10,67 | 0,33 | jasmonate biosynthesis |
| LTR_RH vs. LTR_C | JA | MSTRG.575.1 | MSTRG.575 | -4,4007 | LOX21 | Linoleate 13S-lipoxygenase 2-1 | 4,67 | 0,00 | jasmonate biosynthesis |

| **Comparison** | **Hormone** | **Trasncript ID** | **Gene ID** | **log2FoldChange** | **Symbol** | **Gene description** | **RPKM Transplanted** | **RPKM Container** | **Function** |
| --- | --- | --- | --- | --- | --- | --- | --- | --- | --- |
| MLR_C vs. MLR_CRH | SA | MSTRG.11572.1 | MSTRG.11572 | 1,6568 | EPS1 | Protein ENHANCED PSEUDOMONAS SUSCEPTIBILITY 1 | 226,00 | 787,00 | salicylic acid biosynthesis |
| MLR_C vs. MLR_CRH | SA | MSTRG.32865.2 | MSTRG.32865 | 2,4552 | EPS1 | Protein ENHANCED PSEUDOMONAS SUSCEPTIBILITY 1 | 48,33 | 290,33 | salicylic acid biosynthesis |
| MLR_C vs. MLR_CRH | SA | MSTRG.16782.2 | MSTRG.16782 | 1,9309 | SABP2 | Salicylic acid-binding protein 2 | 384,67 | 1612,67 | salicylic acid biosynthesis |
| MLR_C vs. MLR_CRH | SA | MSTRG.6567.1 | MSTRG.6567 | 2,0693 | SABP2 | Salicylic acid-binding protein 2 | 10,67 | 49,67 | salicylic acid biosynthesis |
| MLR_C vs. MLR_CRH | SA | MSTRG.16784.1 | MSTRG.16784 | 2,1565 | SABP2 | Salicylic acid-binding protein 2 | 30,67 | 150,00 | salicylic acid biosynthesis |
| MLR_C vs. MLR_CRH | SA | MSTRG.34341.1 | MSTRG.34341 | 2,5333 | BSMT2 | S-adenosyl-L-methionine:benzoic acid/salicylic acid carboxyl methyltransferase 2 | 54,00 | 342,33 | salicylic acid biosynthesis |
| MLR_C vs. MLR_CRH | SA | MSTRG.10608.2 | MSTRG.10608 | 3,0625 | BSMT3 | S-adenosyl-L-methionine:benzoic acid/salicylic acid carboxyl methyltransferase 3 | 4,67 | 43,33 | salicylic acid biosynthesis |
| MLR_C vs. MLR_CRH | SA | MSTRG.28611.1 | MSTRG.28611 | 3,3071 | BSMT2 | S-adenosyl-L-methionine:benzoic acid/salicylic acid carboxyl methyltransferase 2 | 3,67 | 39,67 | salicylic acid biosynthesis |
| LLR_C vs. LLR_CRH | SA | MSTRG.32865.2 | MSTRG.32865 | 1,8702 | EPS1 | Protein ENHANCED PSEUDOMONAS SUSCEPTIBILITY 1 | 76,67 | 321,00 | salicylic acid biosynthesis |
| LLR_C vs. LLR_CRH | SA | MSTRG.18629.1 | MSTRG.18629 | 2,3134 | BSMT1 | S-adenosyl-L-methionine:benzoic acid/salicylic acid carboxyl methyltransferase 1 | 61,00 | 344,67 | salicylic acid biosynthesis |
| LLR_C vs. LLR_CRH | SA | MSTRG.28611.2 | MSTRG.28611 | 3,6715 | BSMT3 | S-adenosyl-L-methionine:benzoic acid/salicylic acid carboxyl methyltransferase 3 | 10,67 | 156,33 | salicylic acid biosynthesis |
| LLR_C vs. LLR_CRH | SA | MSTRG.16250.1 | MSTRG.16250 | 5,3258 | BSMT2 | S-adenosyl-L-methionine:benzoic acid/salicylic acid carboxyl methyltransferase 2 | 1,33 | 61,67 | salicylic acid biosynthesis |
| MEZ_C vs. MEZ_CRH | SA | MSTRG.32865.2 | MSTRG.32865 | 1,8432 | EPS1 | Protein ENHANCED PSEUDOMONAS SUSCEPTIBILITY 1 | 711,67 | 2663,67 | salicylic acid biosynthesis |
| STR_C vs. STR_CRH | SA | MSTRG.18629.1 | MSTRG.18629 | 3,3665 | BSMT1 | S-adenosyl-L-methionine:benzoic acid/salicylic acid carboxyl methyltransferase 1 | 6,33 | 50,67 | salicylic acid biosynthesis |
| STR_C vs. STR_CRH | SA | MSTRG.28611.1 | MSTRG.28611 | 3,5283 | BSMT2 | S-adenosyl-L-methionine:benzoic acid/salicylic acid carboxyl methyltransferase 2 | 102,00 | 1117,67 | salicylic acid biosynthesis |
| MTR_C vs. MTR_CRH | SA | MSTRG.18629.1 | MSTRG.18629 | 2,767 | BSMT1 | S-adenosyl-L-methionine:benzoic acid/salicylic acid carboxyl methyltransferase 1 | 9,00 | 66,67 | salicylic acid biosynthesis |
| LTR_C vs. LTR_CRH | SA | MSTRG.10608.2 | MSTRG.10608 | 2,2952 | BSMT3 | S-adenosyl-L-methionine:benzoic acid/salicylic acid carboxyl methyltransferase 3 | 10,67 | 66,00 | salicylic acid biosynthesis |
| **Comparison** | **Hormone** | **Trasncript ID** | **Gene ID** | **log2FoldChange** | **Symbol** | **Gene description** | **RPKM Transplanted** | **RPKM Rhizotron** | **Function** |
| MLR_RH vs. MLR_CRH | SA | MSTRG.16790.2 | MSTRG.16790 | 2,0967 | SABP2 | Salicylic acid-binding protein 2 | 381,33 | 1636,33 | salicylic acid biosynthesis |
| MLR_RH vs. MLR_CRH | SA | MSTRG.16782.2 | MSTRG.16782 | 2,1955 | SABP2 | Salicylic acid-binding protein 2 | 384,67 | 1769,67 | salicylic acid biosynthesis |
| MLR_RH vs. MLR_CRH | SA | MSTRG.16784.1 | MSTRG.16784 | 2,3985 | SABP2 | Salicylic acid-binding protein 2 | 30,67 | 161,67 | salicylic acid biosynthesis |
| MLR_RH vs. MLR_CRH | SA | MSTRG.6567.1 | MSTRG.6567 | 2,4592 | SABP2 | Salicylic acid-binding protein 2 | 10,67 | 59,33 | salicylic acid biosynthesis |
| MLR_RH vs. MLR_CRH | SA | MSTRG.34341.1 | MSTRG.34341 | 3,544 | BSMT2 | S-adenosyl-L-methionine:benzoic acid/salicylic acid carboxyl methyltransferase 2 | 54,00 | 631,33 | salicylic acid biosynthesis |
| MLR_RH vs. MLR_CRH | SA | MSTRG.28611.2 | MSTRG.28611 | 3,6765 | BSMT3 | S-adenosyl-L-methionine:benzoic acid/salicylic acid carboxyl methyltransferase 3 | 3,67 | 46,33 | salicylic acid biosynthesis |
| MLR_RH vs. MLR_CRH | SA | MSTRG.10608.2 | MSTRG.10608 | 3,8565 | BSMT3 | S-adenosyl-L-methionine:benzoic acid/salicylic acid carboxyl methyltransferase 3 | 4,67 | 68,33 | salicylic acid biosynthesis |
| LEZ_RH vs. LEZ_CRH | SA | MSTRG.18629.1 | MSTRG.18629 | 3,117 | BSMT1 | S-adenosyl-L-methionine:benzoic acid/salicylic acid carboxyl methyltransferase 1 | 61,67 | 529,00 | salicylic acid biosynthesis |
| STR_RH vs. STR_CRH | SA | MSTRG.28611.1 | MSTRG.28611 | 2,6592 | BSMT2 | S-adenosyl-L-methionine:benzoic acid/salicylic acid carboxyl methyltransferase 2 | 102,00 | 613,33 | salicylic acid biosynthesis |
| STR_RH vs. STR_CRH | SA | MSTRG.18629.1 | MSTRG.18629 | 3,7462 | BSMT1 | S-adenosyl-L-methionine:benzoic acid/salicylic acid carboxyl methyltransferase 1 | 6,33 | 80,67 | salicylic acid biosynthesis |
| STR_RH vs. STR_CRH | SA | MSTRG.32865.2 | MSTRG.32865 | 2,0331 | EPS1 | Protein ENHANCED PSEUDOMONAS SUSCEPTIBILITY 1 | 570,67 | 2230,67 | salicylic acid biosynthesis |
| MTR_RH vs. MTR_CRH | SA | MSTRG.32865.2 | MSTRG.32865 | 1,8283 | EPS1 | Protein ENHANCED PSEUDOMONAS SUSCEPTIBILITY 1 | 721,67 | 2557,00 | salicylic acid biosynthesis |
| MTR_RH vs. MTR_CRH | SA | MSTRG.18629.1 | MSTRG.18629 | 1,5689 | BSMT1 | S-adenosyl-L-methionine:benzoic acid/salicylic acid carboxyl methyltransferase 1 | 9,00 | 27,00 | salicylic acid biosynthesis |
| MTR_RH vs. MTR_CRH | SA | MSTRG.28611.1 | MSTRG.28611 | 1,574 | BSMT2 | S-adenosyl-L-methionine:benzoic acid/salicylic acid carboxyl methyltransferase 2 | 113,67 | 337,33 | salicylic acid biosynthesis |
| LTR_RH vs. LTR_CRH | SA | MSTRG.16250.1 | MSTRG.16250 | 2,0643 | BSMT2 | S-adenosyl-L-methionine:benzoic acid/salicylic acid carboxyl methyltransferase 2 | 70,00 | 279,33 | salicylic acid biosynthesis |

| **Comparison** | **Hormone** | **Trasncript ID** | **Gene ID** | **log2FoldChange** | **Symbol** | **Gene description** | **RPKM Container** | **RPKM Rhizotron** | **Function** |
| --- | --- | --- | --- | --- | --- | --- | --- | --- | --- |
| LLR_RH vs. LLR_C | SA | MSTRG.16250.1 | MSTRG.16250 | -5,9687 | BSMT2 | S-adenosyl-L-methionine:benzoic acid/salicylic acid carboxyl methyltransferase 2 | 61,67 | 1,00 | salicylic acid biosynthesis |
| LLR_RH vs. LLR_C | SA | MSTRG.29136.1 | MSTRG.29136 | -4,3004 | BSMT3 | S-adenosyl-L-methionine:benzoic acid/salicylic acid carboxyl methyltransferase 3 | 7,00 | 0,33 | salicylic acid biosynthesis |
| LLR_RH vs. LLR_C | SA | MSTRG.28611.1 | MSTRG.28611 | -2,8483 | BSMT2 | S-adenosyl-L-methionine:benzoic acid/salicylic acid carboxyl methyltransferase 2 | 156,33 | 22,00 | salicylic acid biosynthesis |
| MTR_RH vs. MTR_C | SA | MSTRG.16250.1 | MSTRG.16250 | -4,9345 | BSMT2 | S-adenosyl-L-methionine:benzoic acid/salicylic acid carboxyl methyltransferase 2 | 31,67 | 1,00 | salicylic acid biosynthesis |
| MTR_RH vs. MTR_C | SA | MSTRG.34341.1 | MSTRG.34341 | -4,1376 | BSMT2 | S-adenosyl-L-methionine:benzoic acid/salicylic acid carboxyl methyltransferase 2 | 75,67 | 4,33 | salicylic acid biosynthesis |
| MTR_RH vs. MTR_C | SA | MSTRG.6568.3 | MSTRG.6568 | -1,9195 | SABP2 | Salicylic acid-binding protein 2 | 11793,00 | 2992,33 | salicylic acid biosynthesis |
| LTR_RH vs. LTR_C | SA | MSTRG.18629.1 | MSTRG.18629 | 3,4702 | BSMT1 | S-adenosyl-L-methionine:benzoic acid/salicylic acid carboxyl methyltransferase 1 | 21,00 | 194,00 | salicylic acid biosynthesis |
| LTR_RH vs. LTR_C | SA | MSTRG.16250.1 | MSTRG.16250 | 6,8106 | BSMT2 | S-adenosyl-L-methionine:benzoic acid/salicylic acid carboxyl methyltransferase 2 | 3,00 | 279,33 | salicylic acid biosynthesis |
